# Bacterial-Derived Immunomodulators as a Preventive Strategy for Viral Respiratory Tract Infections and Associated Wheezing or Asthma in Children: A Targeted Narrative Review

**DOI:** 10.3390/children13060737

**Published:** 2026-05-26

**Authors:** Manuel E. Soto-Martinez, Wojciech Feleszko, Alexander Moeller

**Affiliations:** 1 Servicio de Neumología, Departamento de Pediatría, Hospital Nacional de Niños “Dr. Carlos Sáenz Herrera”, Caja Costarricense de Seguro Social, San José 1654-1000, Costa Rica; 2 Departamento de Pediatría, Escuela de Medicina, Universidad de Costa Rica, San José 11501-2060, Costa Rica; 3 Department of Pediatric Pneumonology and Allergy, Medical University of Warsaw, 02-091 Warsaw, Poland; wojciech.feleszko@wum.edu.pl; 4 Department of Respiratory Medicine, University Children’s Hospital Zurich, 8032 Zurich, Switzerland; alexander.moeller@kispi.uzh.ch

**Keywords:** immunomodulators, respiratory tract infections, wheezing, pediatric asthma, trained immunity, early-life prevention

## Abstract

**Highlights:**

**What are the main findings?**

- Four bacterial-derived immunomodulators (OM-85, PMBL, MV130, and CRL1505) share convergent mechanisms involving epithelial barrier reinforcement, innate immune activation, and adaptive immune modulation relevant to pediatric respiratory infection prevention.
- Clinical evidence varies substantially across products, with OM-85 demonstrating the most extensive support (showing 26–36% reduction in respiratory tract infections across multiple RCTs and meta-analyses), while data for PMBL, MV130, and CRL1505 remain more limited.

**What are the implications of the main findings?**

- Bacterial-derived immunomodulators represent a promising complementary strategy for reducing recurrent respiratory infections and associated wheezing in young children, alongside standard preventive measures.
- Significant knowledge gaps persist regarding optimal treatment duration, patient selection, and comparative efficacy, highlighting the need for well-designed head-to-head trials with standardized outcomes.

**Abstract:**

Background/Objectives: Respiratory tract infections (RTIs) are a leading cause of morbidity in children under five, with over 75% experiencing recurrent episodes and an increased risk of asthma by school age, particularly following respiratory syncytial virus (RSV) and rhinovirus (RV) infections. While current therapies primarily address acute symptoms, effective preventive strategies remain limited. Bacterial-derived immunomodulators have emerged as promising interventions, but their mechanisms and pediatric clinical evidence remain incompletely characterized. This narrative review examines preclinical mechanisms and clinical findings for four such agents, contextualizing current evidence and identifying key gaps. Methods: A targeted narrative review of PubMed-indexed literature (inception to September 2025) was conducted. Mechanistic studies, pediatric (0–18 years) clinical trials, and meta-analyses evaluating OM-85, polyvalent mechanical bacterial lysates (PMBL/Ismigen), MV130, and *Lactobacillus rhamnosus* CRL1505 were included. Outcomes of interest comprised immunological mechanisms, RTI incidence, wheezing, and asthma-related outcomes. Results: All four agents share convergent immunomodulatory mechanisms involving epithelial barrier reinforcement, innate immune activation, and adaptive immune modulation. OM-85 has the most extensive preclinical evidence. PMBL enhances epithelial repair via the IL-23/IL-22 axis, MV130 induces trained immunity, and CRL1505 acts through the gut–lung axis. Clinical evidence varies markedly, with OM-85 showing the most comprehensive data (18 RCTs and 7 meta-analyses), followed by PMBL and MV130, while evidence for CRL1505 remains predominantly preclinical. Conclusions: Despite variable evidence maturity, these agents share a coherent mechanistic rationale and favorable safety profiles, with ongoing studies expected to clarify their clinical role in early-life respiratory prevention.

## 1. Introduction

Respiratory tract infections (RTIs), particularly those of viral origin such as respiratory syncytial virus (RSV), rhinovirus (RV), influenza virus (IFV), human parainfluenza virus (hPIV), and human metapneumovirus (hMPV), remain among the leading causes of morbidity and mortality in early childhood [1,2,3]. Global estimates for 2019 indicate that RSV alone accounted for 3.6 million acute lower respiratory infection (ALRI) hospitalizations and 101,400 deaths among children under five [4], representing the predominant viral pathogen. Additional substantial burdens are attributed to IFV (0.9 million hospitalizations, 34,800 deaths), hPIV (1 million hospitalizations, 53,000 deaths), and hMPV (0.6 million hospitalizations, 16,100 deaths), with peak mortality concentrated among infants 0–12 months of age [1]. RTI burden persists in resource-rich settings despite advanced medical care. United States surveillance data (2015–2019) demonstrate that RSV causes 58,000–80,000 hospitalizations annually, with the highest incidence among infants under two months (71.6 per 1000; 95% confidence interval (CI), 66.6–76.6) [5]. European Union data (average 2006–2018) report 245,244 yearly RSV-associated hospitalizations in children under five, predominantly (75%) occurring in the first year of life [6]. Between birth and age five years, the immune system undergoes intense maturation while facing continuous pathogen exposure. During this critical period, children exhibit reduced type I interferon (IFN) production, suboptimal dendritic cell (DC) and natural killer (NK) cell activation, restricted T cell repertoire diversity, and cytokine profiles skewed toward type 2 responses [7,8,9]. This immunological immaturity results in heightened susceptibility to respiratory pathogens and exaggerated inflammatory responses that can persist after infection [10], contributing to both high RTI frequency and potential asthma development [11].

Despite the substantial RTI burden, therapeutic management remains limited to supportive care. Bronchodilators and corticosteroids may alleviate acute symptoms but fail to prevent the immune dysregulation linking recurrent infections in young children to chronic respiratory disease [12]. This therapeutic gap has profound long-term consequences: RSV infections in the first year of life are associated with a 30% increase in asthma risk by age 5 years [13], and RV wheezing illness in the first 3 years of life is associated with an increased risk of wheezing/asthma in later life (relative risk (RR) = 2.00, 95% CI 1.62 to 2.49, *p* < 0.001) [14,15]. These epidemiological associations reflect underlying pathogenic mechanisms. Viral infections disrupt epithelial integrity, sustain type 2 inflammation, and attenuate IFN signaling, perpetuating airway hyperreactivity [16]. Each infection compounds immune dysregulation, creating a vicious cycle from acute RTI to chronic airway disease. These limitations underscore an urgent need for host-directed immunomodulatory strategies that enhance mucosal immunity and innate defenses during the critical early-life window. Bacterial-derived immunomodulators have emerged as promising approaches to address this gap. OM-85, polyvalent mechanical bacterial lysate (PMBL), MV130, and *Lactobacillus rhamnosus* CRL1505 exemplify agents that train innate immunity, enhance mucosal defenses, and reduce infection recurrence through diverse immunological mechanisms [17,18,19,20,21,22]. However, persistent uncertainties surrounding bacterial-derived immunomodulators limit clinical integration. Their specific mechanisms of action, pediatric efficacy, and optimal positioning within respiratory care strategies remain poorly defined.

To address these uncertainties, this targeted narrative review aims to synthesize preclinical and clinical evidence supporting the use of four bacterial-derived immunomodulators, OM-85, PMBL, MV130, and *Lactobacillus rhamnosus* CRL1505, in the prevention of pediatric RTI.

Specifically, it seeks to

- Outline the features of the immature immune response in pediatric populations and the pathophysiological mechanisms involved in RTIs and asthma;
- Describe the immunological mechanisms by which bacterial products influence epithelial barrier function, innate and adaptive immunity, and inflammation regulation;
- Summarize their clinical efficacy and safety in reducing RTI incidence and preventing wheezing or asthma outcomes in children;
- Identify remaining evidence limitations and future research priorities to optimize host-directed immunomodulation in pediatric respiratory health.

## 2. Materials and Methods

### 2.1. Selection of Immunomodulators

Four bacterial-derived immunomodulators were selected for this targeted narrative review to represent distinct manufacturing processes and mechanistic paradigms and to meet the following criteria: (1) availability of mechanistic data from preclinical studies investigating modulation of epithelial barrier function, innate immunity, adaptive immunity and inflammation control; (2) documented clinical use or ongoing clinical evaluation in pediatric populations (≤18 years); and (3) distinct production processes covering the main categories of bacterial-derived products. The selected agents were OM-85 (chemical lysis), PMBL/Ismigen (mechanical lysis), MV130/Bactek/Bacmune (heat inactivation), and *Lactobacillus rhamnosus* CRL1505 (live probiotic strain). Other immunomodulatory products were not included because they either lacked sufficient pediatric mechanistic characterization, had limited pediatric respiratory data, or did not represent a distinct biological platform beyond the categories already included in this review.

### 2.2. Literature Search Strategy

Targeted, product-specific searches were performed in PubMed/MEDLINE (inception to September 2025) to map mechanistic and clinical evidence for each product. For mechanistic evidence, product names were combined with terms such as mechanism, immunomodulation, immune response, epithelial barrier, innate immunity, adaptive immunity, in vitro, in vivo, respiratory infection, and asthma. For clinical evidence, searches combined product names with clinical trial, randomized controlled trial (RCT), respiratory infection, asthma, pediatric, or children. Complete search strategies, including exact queries and filters applied, are provided in Supplementary Materials S1. No formal dual independent extraction was performed.

### 2.3. Study Selection and Inclusion Criteria

To provide mechanistic evidence, we included original preclinical studies (in vitro and in vivo) investigating immunological mechanisms relevant to the prevention of respiratory infections. In a heuristic mapping process, each immunomodulator was assigned a preclinical evidence volume category (+++ extensive evidence, ≥20 studies, ++ moderate evidence, 10–19 studies, or + limited evidence, <10 studies) based on the number of mechanistic studies identified; this classification reflects the breadth of the literature rather than certainty or study quality. Complete study characteristics are provided in Supplementary Materials S2: Tables S1–S4.

For clinical evidence, we included (1) RCTs in pediatric populations (≤18 years); (2) systematic reviews and meta-analyses of RCTs; and (3) observational studies when RCT data were limited. We excluded single case reports, studies in exclusively adult populations (>18 years), non-peer-reviewed conference abstracts without full-text publications, studies without respiratory outcomes, and studies indexed outside of PubMed. Complete RCT and meta-analysis study characteristics are provided in Supplementary Materials S2: Tables S5–S7.

### 2.4. Data Synthesis

For preclinical evidence, mechanistic data were synthesized by immune targets, including epithelial barrier function, innate immunity, adaptive immunity, and inflammation regulation, to characterize each product’s immunomodulatory profile. For clinical evidence, we extracted data from published meta-analyses and individual RCTs for each immunomodulatory agent. Evidence was stratified by maturity: extensive (multiple converging meta-analyses), preliminary (positive signals requiring replication), or limited clinical data (mechanistic rationale only). Detailed preclinical evidence and clinical data are provided in Supplementary Materials S2.

### 2.5. Study Design Considerations

This review was designed as a narrative synthesis rather than a formal preregistered systematic review. We used targeted searches to provide an evidence map across mechanistically distinct interventions. This design enables a broad contextualization of both emerging and established interventions while acknowledging the inherent methodological limitations of narrative evidence synthesis.

Based on the targeted literature search and evidence synthesis described above, the following sections summarize and analyze the current mechanistic and clinical evidence regarding bacterial-derived immunomodulators in pediatric respiratory infections, wheezing, and asthma.

## 3. Immunopathophysiology of RTIs and Asthma

During infancy, RTIs are strongly linked to the later development of wheezing and asthma [1,23,24]. The transition from transient viral infection to chronic airway disease reflects complex interactions between immune immaturity, exaggerated inflammation, and impaired epithelial repair [25,26]. During the critical 0–5-year developmental window, these factors may imprint long-term pro-asthmatic immune patterns [27,28]. Understanding this pathophysiological cascade provides the mechanistic rationale for immunomodulatory interventions targeting early-life immune vulnerabilities.

### 3.1. Pediatric Immune Vulnerabilities

The pediatric immune system displays profound structural and functional immaturity throughout the first five years of life [7,29]. Key vulnerabilities include reduced neutrophil bactericidal activity, diminished NK cell cytotoxicity, persistent type 2 bias due to inefficient DC activation, and suboptimal regulatory T cell (Treg) function [7,29]. B lymphocytes mainly produce low-affinity antibodies with delayed class switching, limiting viral neutralization and adaptive memory formation [9,29]. Innate antiviral responses are weak, with reduced type I/III IFN production and impaired epithelial barrier defenses [1,3,25]. This immunological fragility gradually improves through antigen exposure, vaccination, and microbiota maturation [18,30], but, during this vulnerable early-life window, recurrent and severe respiratory infections can trigger long-term airway consequences.

### 3.2. Viral Infection and Dysregulated Host Response

Major respiratory viruses primarily target airway epithelial cells, the first line of defense in the respiratory tract. RSV binds to nucleolin receptors on the epithelial surface [31], RV attaches to intercellular adhesion molecule 1 (ICAM-1) [32], and IFV recognizes sialic acid residues as entry receptors [33]. Viral infection triggers alarmin release (interleukin (IL)-33, thymic stromal lymphopoietin (TSLP), IL-25), which activates DCs, group-2 innate lymphoid cells (ILC2), mast cells, and eosinophils [34]. In parallel, viral nucleic acids engage the pattern recognition receptor (PRR) (toll-like receptor (TLR), retinoic acid-inducible gene I (RIG-I)) to initiate IFN and pro-inflammatory cytokine production (IL-6, tumor necrosis factor alpha (TNF-α)) alongside immune cell recruitment [26]. In the immature pediatric immune system, however, this response becomes pathologically skewed. The pre-existing vulnerabilities described above—weak IFN responses, insufficient antibody maturation, and T helper type 2 (Th2)/ILC2 dominance—converge to amplify viral pathogenesis [7]. Excessive neutrophil recruitment and type 2 cytokines (IL-4, IL-5, IL-13) promote epithelial injury, mucus hypersecretion, and early airway remodeling [28,35]. Viral–bacterial co-infections (*Moraxella catarrhalis*, *Haemophilus influenzae*, *Streptococcus pneumoniae*) further amplify inflammation and worsen disease severity [25], impairing mucociliary clearance and prolonging inflammatory responses that contribute to recurrent wheezing and early-onset asthma [24].

### 3.3. Mechanisms of Chronic Inflammation and Asthma Development

RTIs occurring during the 0–5-year window can establish persistent inflammatory programs promoting asthma [25,27]. In infants, immune immaturity combined with excessive antiviral activation during severe infections results in high viral loads, prolonged epithelial injury, and sustained airway inflammation [26,36]. Epidemiological data are consistent with this mechanistic framework. For example, cohort studies report that approximately 54% of infants experience RSV infection during the first year of life, which has been associated with a 35% higher risk of asthma by age five, while avoidance of early RSV infection has been linked to a 25% lower asthma risk. In addition, wheezing associated with rhinovirus infection after the first year of life is among the strongest epidemiological predictors of subsequent asthma development [13,15,37]. Risk is particularly elevated in children with atopic backgrounds, where viruses act as inflammatory triggers of genetically primed susceptibility [23,27]. Whether prevention of RSV infection or severe RSV disease reduces long-term asthma risk remains an active area of investigation. Pediatric asthma displays heterogeneous inflammatory patterns at the cellular level. Some children exhibit persistent neutrophilic inflammation with elevated IL-8, chemokine CXCL10, granulocyte colony-stimulating factor (G-CSF), and IL-17 even during infection-free periods [28], contributing to epithelial damage and mucus retention. Others show mixed T helper type 1/T helper type 2 (Th1/Th2) activation, perpetuating inflammatory cycles: Th2 cytokines drive allergic sensitization and increase infection susceptibility, while repeated viral episodes reinforce Th1 inflammation [26,35]. Sustained ILC2 activation maintains IL-13 production, promoting mucus hypersecretion, goblet cell metaplasia, and airway smooth muscle contraction [34,35]. These cellular mechanisms converge to drive structural airway changes.

### 3.4. Airway Remodeling and Structural Changes

Building on these inflammatory mechanisms, repeated viral infections progressively remodel airway structure through airway wall thickening, increased smooth muscle mass, subepithelial fibrosis, and mucus gland hyperplasia, reducing airway caliber and increasing obstruction [26,28,36]. These structural changes compromise elasticity, rendering children prone to obstruction during subsequent infections or allergen exposures. Preschool children with recurrent wheezing already display increased airway smooth muscle mass, strongly associated with asthma persistence [38,39,40]. Recurrent RTIs and prolonged inflammation during early lung development alter growth trajectories, leading to reduced maximal lung capacity persisting into adulthood [14,36]. Individuals with childhood wheezing or asthma history show accelerated lung function decline and markedly higher chronic obstructive pulmonary disease (COPD) risk later in life, especially when childhood asthma is severe or steroid-dependent [36].

### 3.5. Microbiome and Susceptibility to RTIs and Asthma Development

The gut–lung axis bidirectionally influences immune maturation and respiratory health [22,41]. During early life, disturbances in microbial composition, such as reduced diversity, depletion of protective commensals (*Bifidobacterium*, *Lactobacillus*), and overgrowth of pro-inflammatory taxa, impair the host’s capacity to maintain immune balance [30]. These microbial imbalances alter respiratory immunity through three main mechanisms: (1) disruption of microbial metabolite production, especially short-chain fatty acids (SCFA) that regulate epithelial and Treg function; (2) impaired DC maturation; and (3) dysregulated systemic cytokine signaling [22]. Gut dysbiosis during the 0–5 years immune maturation window increases asthma risk by interfering with immune education [18,30]. High-risk infants often exhibit reduced Lachnospira and Faecalibacterium alongside elevated *Streptococcus* and *Bacteroides*, fostering pro-inflammatory environments [22,41]. Such dysbiosis predisposes children to severe viral infection symptoms and facilitates transition from recurrent wheezing to chronic asthma by sustaining mucosal inflammation.

### 3.6. Genetic and Environmental Factors

Genetic variants in immune response genes (STAT4, MX1) increase vulnerability to severe viral infections and asthma by disrupting IFN signaling and antiviral defenses [42,43]. Environmental exposures compound this genetic susceptibility: tobacco smoke and air pollution impair mucociliary clearance, induce oxidative stress, and sustain low-grade inflammation, collectively enhancing infection severity and asthma risk [3,43,44]. This gene environment interaction creates self-perpetuating cycles. Genetically predisposed children experience severe viral infections that damage airway epithelium, increase pollutant sensitivity, and further compromise immune defenses and epithelial repair. Each infection–exposure cycle deepens remodeling and chronic inflammation, shifting disease trajectory from episodic wheezing to persistent asthma [42,44]. It is important to note that future trials should consider stratification by family history of asthma or atopy, atopic sensitization, tobacco smoke exposure, air pollution exposure, and other environmental modifiers, as these factors may influence both baseline risk and response to immunomodulatory interventions.

### 3.7. Implications for Immunomodulatory Strategies

The pathophysiological cascade described above reveals a critical therapeutic window during immune system maturation (0–5 years). Successful immunomodulation must simultaneously: (1) compensate for weak antiviral responses; (2) correct Th1/Th2 imbalances; (3) enhance regulatory mechanisms; and (4) prevent transition from acute inflammation to chronic remodeling. The following section explores how bacterial-derived immunomodulators act to mitigate these immune vulnerabilities.

## 4. Immunomodulation Agents and Mechanisms of Action

**Figure 1:**
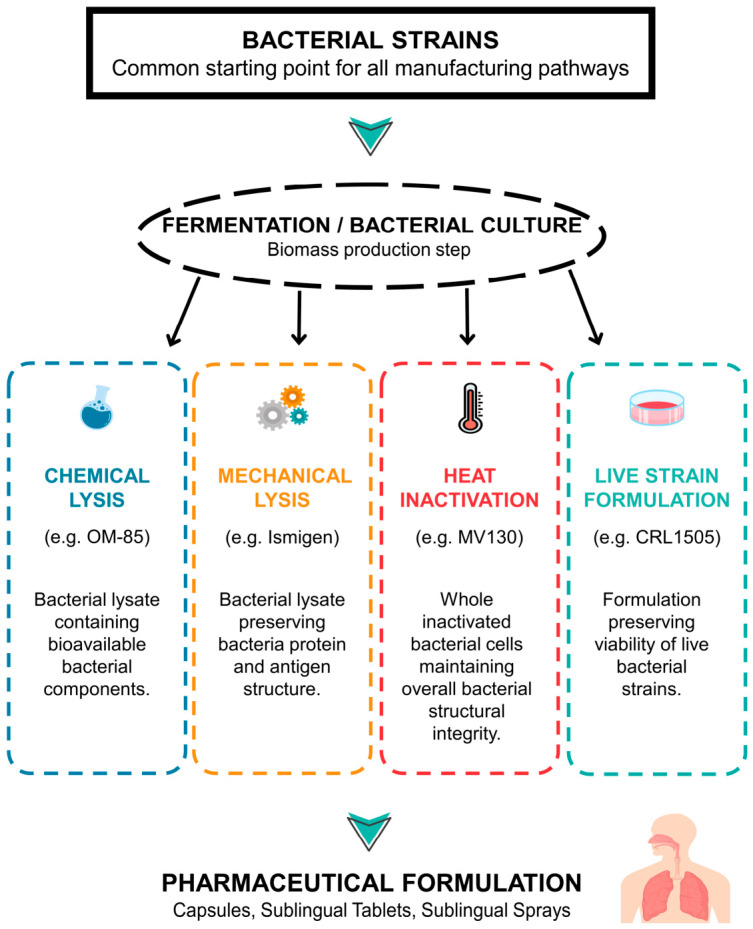
Schematic representation of bacterial strain manufacturing pathways leading to different formulations. Bacterial strains serve as the common starting point for all manufacturing pathways of immunomodulatory preparations. Four main processes can be applied: chemical lysis (e.g., OM-85 [46]), mechanical lysis (e.g., PMBL/Ismigen [47]), heat inactivation (e.g., MV130 [21,48]), and live strain formulation (e.g., CRL1505 [49]). Other processing methods may exist but are less commonly employed for respiratory immunomodulators. This is a conceptual diagram; actual manufacturing processes may include additional quality control and purification steps. Abbreviations: PMBL, polyvalent mechanical bacterial lysate; MV130, mucosal vaccine 130; CRL1505, *Lactobacillus rhamnosus* CRL1505.

Immunomodulators are promising strategies to prevent recurrent RTIs in early life [20,39,45]. Among them, bacterial-derived immunomodulators have been extensively studied. Their composition and immunological imprint vary according to the manufacturing process, as shown in Figure 1:

- Chemical lysis through alkaline treatment generates bacterial lysate enriched in bioavailable protein, peptide, lipoteichoic acids, and detoxified lipopolysaccharides, compounds approaching metabolic end-products [46].
- Mechanical lysis through high-pressure disruption or sonication generates bacterial lysate containing structural fragments and soluble components, bacteria protein, and antigen structures, while ensuring the absence of viable bacteria [47].
- Heat inactivation of whole-cell bacteria through heat or formalin treatment maintains overall bacterial structural integrity while ensuring non-viability [21,48].
- Live strain formulations maintain viability and colonization capacity [49].

**Figure 2:**
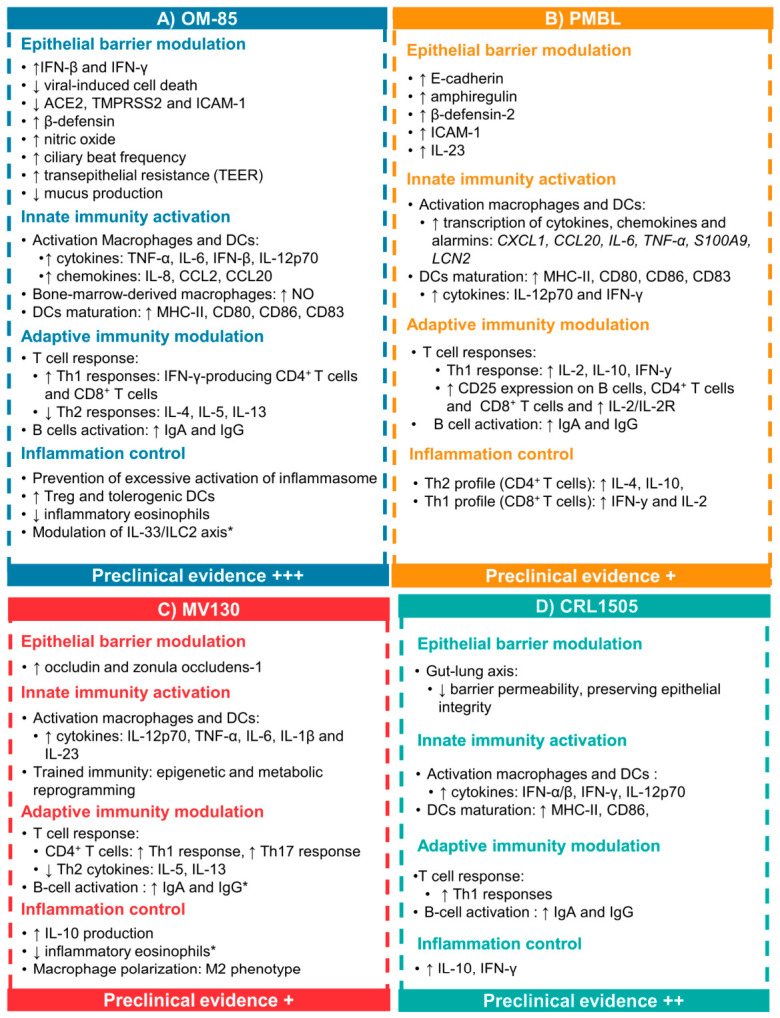
Overview of immunomodulatory mechanisms of OM-85, PMBL, MV130, and CRL1505. The figure summarizes the main immunomodulatory mechanisms that were published in the literature of four bacterial-derived preparations, highlighting their ability to strengthen epithelial barriers, activate innate defenses, modulate adaptive immunity and control inflammation. (**A**) OM-85 demonstrates extensive preclinical evidence across epithelial [50,51,52,53,54,55], innate, adaptive [56,57,58,59], and anti-inflammatory pathways [53,54,56,60,61,62,63]. (**B**) PMBL modulates epithelial barrier [64] function notably via the IL-23/IL-22 axis and activates both innate and adaptive immune responses [47,65,66,67,68]. (**C**) MV130 induces trained immunity through epigenetic and metabolic reprogramming of innate immune cells [69,70,48,71,72]. (**D**) CRL1505 acts primarily through the gut–lung axis, enhancing mucosal immunity and modulating inflammatory responses [73,74,75,76,77,78,79]. Each immunomodulator was assigned a preclinical evidence level based on the number of mechanistic studies identified: +++ (extensive evidence, ≥20 studies), ++ (moderate evidence, 10–19 studies), or + (limited evidence, <10 studies). Asterisk (*) denotes data obtained through experimental models employing administration routes that differ from the clinical route of administration (e.g., intranasal administration). Abbreviations: ACE2, angiotensin-converting enzyme 2; CCL, chemokine (C-C motif) ligand; CD, cluster of differentiation; CXCL, chemokine (C-X-C motif) ligand; DCs, dendritic cells; ICAM-1, intercellular adhesion molecule 1; IFN, interferon; IgA, immunoglobulin A; IgG, immunoglobulin G; IL, interleukin; IL-2R, interleukin-2 receptor; ILC2, group-2 innate lymphoid cells; LCN2, lipocalin 2; M2, M2 macrophage phenotype; MHC-II, major histocompatibility complex class II; NO, nitric oxide; S100A9, S100 calcium-binding protein A9; TEER, transepithelial electrical resistance; Th1/Th2/Th17, T helper type 1/2/17; TMPRSS2, transmembrane serine protease 2; TNF-α, tumor necrosis factor alpha; Treg, regulatory T cells. Up arrow means increase and Down arrow means decrease.

The following section summarizes the effects of each product on the immune system (epithelial barrier, innate immunity, adaptive immunity, inflammation control) that were reported in the literature, as summarized in Figure 2. Complete study characteristics are provided in Supplementary Materials S2: Tables S1–S4.

### 4.1. Polyvalent Chemical Lysate: OM-85

OM-85 is a lyophilized bacterial extract derived from eight respiratory pathogens: *Haemophilus influenzae*, *Streptococcus pneumoniae*, *Streptococcus pyogenes*, *Streptococcus viridans/sanguinis*, *Staphylococcus aureus*, *Klebsiella pneumoniae* (ssp. *pneumoniae* and ssp. *ozaena*), and *Moraxella catarrhalis*. OM-85 contains readily bioavailable proteins, peptides, lipoteichoic acids, and detoxified lipopolysaccharides, components close to end-stage metabolic products that undergo minimal or no further metabolization [56,60,80]. OM-85 is administered orally for the prevention of recurrent RTIs in children, starting from 6 to 12 months of age depending on national regulations [80].

- Epithelial barrier modulation: In human bronchial epithelial cells, OM-85 enhances mucosal defense through multiple pathways. First, it strengthens antiviral immunity by increasing production of IFN-β and IFN-γ while reducing viral-induced cell death [50,51]. Second, OM-85 interferes with viral entry mechanisms. It prevents the increase in ICAM-1 expression [50,51] and reduces angiotensin-converting enzyme 2 (ACE2) and transmembrane serine protease 2 (TMPRSS2) expression [81,82], thereby limiting viral adhesion while increasing β-defensin, enhancing mucosal protection against pathogens [50,51]. Additionally, in human sinonasal epithelial cells, OM-85 increases nitric oxide production (NO) [52] and ciliary beat frequency [52], supporting mucociliary clearance, and enhances transepithelial resistance (TEER) in human bronchial epithelial cells [53], collectively strengthening epithelial barrier integrity. In murine asthma models, oral OM-85 also prevents airway remodeling by reducing goblet cell hyperplasia and mucus hypersecretion, key indicators of epithelial dysfunction, thereby restoring normal mucosal architecture [54,55].
- Innate immunity activation: In human blood immune cells, OM-85 activates DCs and macrophages through PRR, predominantly TLR4 and TLR2, with contributions from TLR7 and TLR9 [57,58]. This stimulation triggers a MyD88-dependent activation of key innate signaling pathways, including nuclear factor kappa B (NF-κB), TANK-binding kinase 1 (TBK1), mitogen-activated protein kinases (MAPKs), and mammalian target of rapamycin complex 1 (mTORC1), leading to the secretion of major pro-inflammatory and antiviral cytokines such as TNF-α, IL-6, IL-12p70, and IFN-β [57,58]. OM-85 also induces the production of chemokines such as CXCL8, CCL2, and CCL20, facilitating the recruitment and activation of innate immune cells [57,58]. In murine bone marrow-derived macrophages, OM-85 also induces NO production together with NF-κB nuclear translocation, confirming macrophage activation and antimicrobial effector induction [56]. In parallel, DCs exposed to OM-85 also increase the expression of major histocompatibility complex class II (MHC-II), cluster of differentiation 80 (CD80), CD86, and CD83, enhancing antigen presentation capacity and subsequent T cell activation [57,59,60].
- Adaptive immunity modulation: OM-85 promotes a Th1-oriented immune response [56], increasing IFN-γ-producing cluster of differentiation 4 (CD4)^+^ T cells and virus-specific CD8+ T cells [60,61,62,63]. Simultaneously, it prevents the secretion of Th2 cytokines (IL-4, IL-5, IL-13) [54,56]. OM-85 also enhances polyclonal B cell activation, boosting the production of immunoglobulin A (IgA) and immunoglobulin G (IgG) antibodies [56,60], which provide enhanced humoral immunity against respiratory pathogens.
- Inflammation control: OM-85 activates protective immune pathways while preventing excessive inflammasome activation [17]. Following oral administration, OM-85 induces tolerogenic dendritic cells and promotes Treg accumulation in the lungs [54,55], contributing to IL-10-mediated immune homeostasis and decreased eosinophilic inflammation [54,55]. In intranasal administration models, OM-85 specifically modulates the epithelial IL-33/ILC2 axis, further contributing to reduced type 2 inflammation [53].

Through these immunomodulatory mechanisms, OM-85 trains the immune system. Immune training refers to the process of enhancing immune responses to establish an antiviral and antibacterial state leading to a decreased susceptibility to infections, while, in the case of infection, maintaining a balance between robust defenses against pathogens and minimizing damage to healthy tissues by controlling inflammation [17,50,51,52,53,54,55,56,57,58,59,60,61,62,63,81,82,83].

### 4.2. Polyvalent Mechanical Lysate (PMBL)

Among PMBL, Ismigen^®^ is the most widely known formulation. It is a sublingual tablet containing bacterial components derived from *Haemophilus influenzae*, *Streptococcus pneumoniae*, *Streptococcus pyogenes*, *Klebsiella pneumoniae*, *Klebsiella ozaena*, *Staphylococcus aureus*, *Streptococcus viridans*, and *Moraxella catarrhalis* [67,84]. PMBLs are produced by high-pressure mechanical disruption or sonication, processes that preserve bacterial protein and antigen structures while ensuring the absence of viable bacteria [67]. PMBL is administered as sublingual tablets for the prevention of recurrent RTIs in children.

- Epithelial barrier modulation: PMBL directly targets airway epithelial cells, enhancing mucosal barrier integrity. In primary human bronchial epithelial cells, PMBL markedly increases E-cadherin, a tight junction protein critical for maintaining epithelial integrity, amphiregulin, an autocrine growth factor supporting epithelial repair, and β-defensin-2, an antimicrobial peptide conferring direct bacteriostatic activity against pathogens [64]. PMBL also increases ICAM-1 expression [64], which acts as an adhesion molecule mediating leukocyte recruitment and strengthening epithelial–immune crosstalk [64]. Additionally, PMBL stimulates epithelial secretion of IL-23, which in turn triggers type 3 innate lymphoid cell (ILC3)-dependent IL-22 production, a pathway responsible for epithelial repair and antimicrobial peptide release [64], supporting mucosal barrier maturation.
- Innate immunity activation: PMBL activates innate immunity through engagement of PRR on DCs and macrophages, triggering NF-κB signaling in myeloid differentiation primary response 88 (MyD88)-dependent manner [65]. This initiates a transient pro-inflammatory transcriptional program involving cytokines, chemokines and alarmins (CXCL1, CCL20, IL6, TNFA, S100A9, LCN2) that recruit innate effectors to mucosal sites [65]. At the cellular level, PMBL drives maturation of monocyte-derived DCs, increasing the expression of CD80, CD86, CD83, and MHC-II [47]. DCs stimulated with PMBL markedly enhance the secretion of IL-12p70, activate NK cells, and amplify their IFN-γ production and early antimicrobial cytotoxicity [47,66].
- Adaptive immunity modulation: PMBL treatment induces robust IL-12p70 secretion driving Th1 polarization [47], enhancing IFN-γ and IL-2 secretion. In parallel, PMBL upregulates CD25 expression on B cells, CD4^+^ and CD8^+^ T cells, amplifying IL-2/IL-2R signaling pathways essential for lymphocyte proliferation and effector differentiation [67]. This synergistically supports B cell activation, class switching, and plasma cell differentiation [67], culminating in enhanced mucosal IgA secretion [47], which serves as a critical first-line barrier against respiratory pathogens.
- Inflammation control: PMBL balances immune activation with regulation, preventing pathological hyperinflammation. In vitro, PMBL-primed CD4^+^ T cells exhibit a predominantly Th2 profile with markedly elevated IL-4 and IL-10 production, and CD8^+^ T cells adopt a cytotoxic Th1 phenotype (IL-2, IFN-γ), indicating balanced Th1/Th2 activation [67,68]. The protective effect of PMBL occurs independently of neutrophils, IL-17A, or caspase-1, suggesting engagement of multiple parallel effector pathways that prevent over-reliance on single inflammatory axes prone to immunopathology [65].

### 4.3. Heat-Inactivated Whole Bacteria: MV130

MV130 is a sublingual spray containing a suspension of heat-inactivated whole-cell bacteria, including *Haemophilus influenzae*, *Streptococcus pneumoniae*, *Klebsiella pneumoniae*, *Staphylococcus aureus*, *Staphylococcus epidermidis*, and *Moraxella catarrhalis* [70]. MV130 preserves intact bacterial cell structures including cell walls, membranes, and surface proteins, potentially providing broad antigenic stimulation [70]. Most mechanistic data derive from intranasal administration in animal models, but the immunological pathways identified are consistent with the effects observed after clinical sublingual use.

- Epithelial barrier modulation: MV130 reduced barrier disruption markers in bronchoalveolar lavage, increased the expression of tight-junction proteins such as occluding and zonula occludens-1 (ZO-1), and prevented mucus hypersecretion. These effects were associated with decreased airway resistance and smooth muscle thickening, indicating protection of the epithelial barrier [69].
- Innate immunity activation: MV130 triggers dual PRR signaling in myeloid cells, including macrophages and dendritic cells, through TLR/MyD88 and NLR/RIPK2 (receptor-interacting protein kinase 2) pathways, leading to NF-κB activation and the subsequent release of IL-12p70, TNF-α, IL-6, IL-1β, and IL-23 [70]. MV130 has been characterized as a trained-immunity-based vaccine [48], capable of inducing long-term functional reprogramming of myeloid progenitors in the bone marrow. This training is associated with epigenetic remodeling that increases chromatin accessibility at inflammatory genes and metabolic rewiring toward enhanced oxidative phosphorylation (OXPHOS) and glycolysis in an mTOR-dependent manner [48]. These adaptations heighten responsiveness to secondary stimuli, resulting in broad, nonspecific protection. In vivo, trained innate responses induced by MV130 enhance resistance to respiratory viral infections and improve the immunogenicity of unrelated vaccines [71]. Epigenetic characterization of MV130-induced trained immunity is based predominantly on adult and murine models; dedicated pediatric immunophenotyping data on the durability of these changes are currently lacking.
- Adaptive immunity modulation: MV130-activated DCs drive CD4^+^ T cell differentiation toward Th1 (IFN-γ-mediated antiviral) and T helper type 17 (Th17) (IL-17-mediated antibacterial) phenotypes, together with the induction of IL-10-secreting Treg, supporting a balanced and protective adaptive profile [70]. Following intranasal administration, MV130 enhanced mucosal adaptive responses, characterized by increased airway secretory IgA and a Th1-associated antibody profile, reflected by an elevated IgG2c/IgG1 ratio [71]. These effects are consistent with an innate immune reprogramming environment in which trained macrophages and dendritic cells display enhanced responsiveness to secondary challenges, thereby promoting durable mucosal protection [48].
- Inflammation control: MV130 establishes balanced immune responses by activating pro-inflammatory pathways while simultaneously inducing IL-10-dependent regulatory mechanisms [48,70]. This dual action leads to a controlled inflammatory response that resolves once the pathogen threat subsides, while imprinting long-term functional reprogramming of innate immune cells that enhances responsiveness to future challenges. In experimental asthma models, MV130 administered intranasally reduced allergen-specific immunoglobulin E (IgE), attenuated Th2-associated cytokines and eosinophilic infiltration, and prevented airway remodeling, consistent with a protective modulation of allergic inflammation [69]. Additionally, human mesenchymal stromal cells exposed to MV130 promoted macrophage polarization toward an anti-inflammatory M2 phenotype and the generation of regulatory dendritic cells, supporting tissue repair and immune homeostasis [72].

### 4.4. Probiotic: Lactobacillus rhamnosus CRL1505

CRL1505 is a Gram-positive lactic acid bacterium originally isolated from goat’s milk in Argentina and subsequently characterized as an “immunobiotic” strain due to its capacity to modulate systemic and mucosal immune responses beyond the intestinal tract [73,85,86]. Clinically, CRL1505 is administered orally as a fermented dairy product at a typical dose of 10^8^–10^9^ colony-forming units (CFUs) daily and is indicated from infancy for immune support and the prevention of recurrent respiratory infections [86]. The mechanistic rationale rests on the gut–lung axis, where immune signaling from gut-associated lymphoid tissue influences distant mucosa, including the respiratory epithelium [74,75,86].

- Epithelial barrier modulation: CRL1505 strengthens epithelial integrity at both intestinal and respiratory levels through coordinated mucosal crosstalk. Oral administration enhances the intestinal barrier and stimulates immune mediators that influence distant mucosal sites, illustrating a functional gut–lung axis [73,74,76]. This bidirectional communication promotes the production of cytokines such as IL-10 and IFN-β, supporting epithelial repair and IgA secretion in both tissues [73,76]. In the respiratory tract, CRL1505 or its purified peptidoglycan (PG) reduces bronchoalveolar barrier permeability, as shown by lower protein leakage and preserved epithelial morphology in viral-like inflammation models [77].
- Innate immunity activation: Oral CRL1505 enhances respiratory innate antiviral defenses through the activation of intestinal and pulmonary dendritic cells (DCs) and macrophages. In the gut, CRL1505 stimulates DCs to upregulate MHC-II, CD86, and IL-12p70, promoting Th1-oriented responses that extend to the respiratory mucosa via gut–lung immune communication [73,74,76]. In the lungs, macrophages primed by CRL1505 increase the production of type I interferons (IFN-α/β) and IFN-γ, creating an interferon-centered antiviral program that accelerates viral clearance while limiting excessive inflammation [78,79,86].
- Adaptive immunity modulation: CRL1505 strengthens adaptive immune responses in the respiratory tract by promoting Th1 polarization and enhancing mucosal antibody production. Oral administration increases intestinal and pulmonary CD3^+^CD4^+^IFN-γ^+^ T cells through IL-12p70-producing dendritic cells, leading to a Th1-oriented environment that supports antiviral protection [73,74,76]. In parallel, the purified PG derived from CRL1505 enhances B cell recovery and mucosal IgA production, amplifying the humoral arm of adaptive immunity [77,79]. Together, these effects demonstrate that CRL1505 reinforces both cellular and humoral responses essential for long-term protection of the respiratory mucosa.
- Inflammation control: Although CRL1505 activates strong mucosal immune responses, it simultaneously maintains inflammatory balance through the induction of IL-10 in both the lung and systemic compartments [73,74]. This regulatory cytokine counteracts excessive production of pro-inflammatory mediators such as TNF-α, IL-6, and CCL2, thereby limiting tissue injury while preserving efficient pathogen clearance [76,78]. In models of viral and pneumococcal infection, the concurrent upregulation of IFN-γ and IL-10 is associated with reduced lung inflammation and improved survival [73,79]. These findings indicate that CRL1505 promotes a well-orchestrated immune response, combining antiviral efficacy with controlled inflammation to sustain mucosal homeostasis.

## 5. Pediatric Clinical Evidence

Bacterial-derived immunomodulators have been evaluated across a spectrum of clinical development stages, from extensive multicenter trials to retrospective studies. The number of RCTs and meta-analyses identified for each product is summarized in Table 1, revealing substantial heterogeneity in evidence maturity (see Supplementary Materials S1 for search strategies and Supplementary Materials S2: Tables S5–S7 for complete study characteristics). These studies collectively support the inclusion of bacterial products as a key component of preventive strategies for pediatric respiratory health.

### 5.1. Evidence from Clinical Studies

We present clinical evidence stratified by evidence maturity to facilitate transparent interpretation and appropriate clinical application.

#### 5.1.1. Extensive Evidence Supporting Clinical Integration: Polyvalent Chemical Lysate (OM-85)

OM-85 has been evaluated in seven meta-analyses in pediatric populations [87,88,89,90,91,92,93]. Two recent meta-analyses using complementary methodological approaches have evaluated OM-85 efficacy. A conventional meta-analysis (2021) reported a mean difference of 1.16 RTIs (95% CI, −1.66 to −0.65), in favor of OM-85, with significant reductions in antibiotic use and a favorable safety profile [89]. A model-based meta-analysis (2022) employed simulation analysis to estimate absolute infection rates, reporting 5.28 RTIs annually (95% CI: 4.86–5.71) in treated children versus 7.90 (95% CI: 6.83–8.98) in placebo groups. No significant increase in drug-related adverse events was observed (RR: 1.31; 95% CI: 0.54–3.19) [93]. In addition, De Boer et al. reported a significant reduction in both wheezing episodes and asthma exacerbations in pediatric populations, with pooled mean differences of -1.31 (95% CI −2.00 to −0.62; *p* = 0.0002), suggesting additional benefits in inflammatory airway disorder [88]. In line with these studies, the meta-analyses of Yin et al., which included a larger number of studies—53 RCTs involving 4851 children, most of them in the Chinese language—reported a positive effect of OM-85 in terms of frequency of RTIs and decrease in infection and wheezing duration. Beyond RTI frequency, OM-85 improved related clinical outcomes, including antibiotic use and symptoms such as fever, cough, and wheezing. Adverse events were predominantly mild and comparable to a placebo [90].

These results reinforce the conclusions of the Cochrane review [92], which evaluated various immunostimulants and showed that OM-85 reduced the incidence of acute respiratory infections in at-risk children by nearly 40%, without increasing treatment-related adverse events. Together, this progressive accumulation of evidence highlights the robustness, reproducibility, and clinical relevance of OM-85’s preventive efficacy in pediatric respiratory health.

The meta-analyses presented above are based on the body of RCTs (see Supplementary Materials S2). Among these, a single-blind RCT involving 68 children aged 36–59 months with recurrent RTIs is worth highlighting because it provides detailed and clinically relevant information on both the efficacy and safety of OM-85 in the context of co-vaccination with an inactivated influenza vaccine (IIV). In this study, IIV plus OM-85 administered for 3 months significantly reduced the proportion of children experiencing at least one upper RTI or one lower RTI during follow-up compared with IIV only (20% vs. 31%; *p* < 0.05 and 5% vs. 15%; *p* < 0.05, respectively). Additional benefits included reduced school-absence days and antibiotics courses [101].

Real word evidence confirms that OM-85 reduces infection-related morbidity, healthcare visits, and antibiotic use in children with a history of recurrent RTIs [115,116].

Based on the convergence of multiple meta-analyses, individual large-scale RCTs, and real-world data, OM-85 has the most mature clinical evidence base among the products reviewed and may be considered in selected children suffering from recurrent RTIs and wheezing. Five ongoing trials spanning phase II–IV (n = 2308) will further validate these findings across different age groups (infants to preschoolers), clinical contexts (prevention of recurrent RTI and/or wheezing), and follow-up durations (6 months to 5 years) (see detailed in “Section 5.2. Ongoing Research and Future Directions”).

#### 5.1.2. Preliminary Evidence Requiring Expansion: PMBL (Ismigen) and MV130 (Bactek/Bacmune)

The efficacy of PMBL (Ismigen) in reducing the frequency and severity of recurrent RTIs in children has been evaluated in a meta-analysis of 15 RCTs, including three RCTs on pediatric populations, and demonstrated significant RTI reduction in children compared with a placebo (MD of −2.20 episodes, *p* < 0.0001) [19], confirming its protective effect across multiple studies. Clinical trials also support benefits, as shown in five RCTs [110,111,112,113,114]. In a double-blind, placebo-controlled, multicenter trial of 152 children (6–16 years) with recurrent RTIs and partially controlled or uncontrolled allergic asthma, PMBL did not significantly improve the primary endpoint of asthma control but demonstrated significant benefits for the secondary endpoint of asthma exacerbations [111]. At Week 12, the mean number of exacerbations was lower in the PMBL group compared with a placebo (0.3 ± 0.6 vs. 0.8 ± 1.1; *p* = 0.009) [111]. Over the total study period, the PMBL group also had fewer exacerbations (1.1 ± 1.3 vs. 1.9 ± 2.0; *p* = 0.01), fewer days with exacerbation (13.3 ± 11.2 vs. 19.8 ± 15.7; *p* = 0.009), and prolonged time to subsequent exacerbations (hazard ratio (HR) = 0.45 for the second; HR = 0.26 for the third; both *p* < 0.01). Importantly, no serious adverse events related to PMBL were reported [111]. The five individual pediatric RCTs identified (n = 420 total) provide preliminary validation, but larger multicenter trials with longer follow-up are needed to establish definitive efficacy across diverse populations and clinical phenotypes.

MV130 (Bactek/Bacmune) shows a similar evidence profile, characterized by a clear efficacy signal but limited replication. In a phase III randomized, double-blind, placebo-controlled trial including 120 children under 3 years of age with recurrent wheezing, MV130 significantly reduced the frequency of wheezing episodes over a 12-month follow-up. The median annual number of wheezing episodes decreased from 5.0 at baseline to 3.0 following MV130 treatment, corresponding to a 40% reduction compared with a placebo (*p* < 0.001) [102]. Secondary outcomes included shorter wheezing episodes, reduced symptom severity and lower medication use, with no treatment-related adverse events reported [20]. Consistent results were observed in a retrospective cohort of 186 children aged 5 months to 18 years, in whom MV130 prophylaxis for 3–6 months was associated with a 75% reduction in infectious episodes compared with the preceding year. The median number of annual infectious episodes decreased from 5.0 before treatment to 1.0 after treatment (*p* < 0.001), with lower respiratory tract infections declining from a median of 5.0 to 2.0 episodes [21]. These real-world data, while valuable, are limited by the absence of standardized follow-up and potential co-interventions that may confound treatment effects [21]. However, the evidence base currently rests on a single multicenter RCT (n = 120) and retrospective data. Replication in independent cohorts, evaluation across broader age ranges, and head-to-head comparative trials are necessary to establish MV130’s position within evidence-based respiratory care algorithms. Future trials should standardize definitions of recurrent RTI and include head-to-head comparisons to clarify positioning among available bacterial-derived products. PMBL (Ismigen) and MV130 have shown preliminary clinical benefits, yet their validation still depends on larger and more robust multicenter trials.

#### 5.1.3. Strong Mechanistic Rationale, Limited Clinical Data: *Lactobacillus rhamnosus* CRL1505

Unlike the bacterial-derived immunomodulator, CRL1505 currently lacks dedicated pediatric RTI trials despite substantial mechanistic evidence supporting its immunomodulatory potential. This gap reflects the earlier developmental stage of this agent rather than an absence of biological plausibility. General evidence for probiotics in RTI prevention comes from broader pediatric studies involving multiple strains. A systematic review of 14 pediatric RCTs [49] reported that most high-quality studies found reductions in RTI incidence, antibiotic prescriptions, and school absenteeism, with no serious adverse events. Specific strains, such as *Lactobacillus rhamnosus* GG and *Lactobacillus casei* DN-114001, showed significant benefits, including fewer URTIs and acute otitis media episodes [117]. By contrast, an earlier systematic review of 14 RCTs highlighted substantial heterogeneity in study design and outcomes, concluding that probiotics did not consistently reduce RTI incidence [118]. These mixed findings emphasize that probiotic efficacy is highly strain-specific, and evidence from other Lactobacillus strains cannot be directly extrapolated to CRL1505. Although mechanistic evidence and the designation of live probiotics as a distinct product category provide compelling rationale for clinical trials, strong pediatric data on respiratory tract infections remain scarce. The ongoing NCT07154992 trial (detailed below) is expected to address this critical evidence gap.

### 5.2. Ongoing Research and Future Directions

The 2024 European Respiratory Society (ERS) statement on preschool wheezing emphasizes phenotyping/treatable traits and identifies immunomodulators as an area of focus. It highlights promising immunomodulatory interventions, particularly for children suffering from recurrent RTIs and wheezing episodes. However, the task force emphasizes the need for further research to address critical clinical gaps, including evaluation in diverse populations (patients with a wheezing history, high-risk groups), long-term studies, and the identification of biomarkers for implementation in general practice. To address these needs, bacterial products are currently under investigation in large, multicenter randomized controlled trials with distinct aims, which will significantly expand and refine the clinical evidence base. The ongoing studies are presented in Table 2.

OM-85: Efficacy in recurrent RTIs and Asthma Prevention: Five ongoing phase II–IV trials are evaluating OM-85 in pediatric populations [119,120,121,122,123]. We summarize below three representative studies that exemplify the key research questions being addressed, specifically focusing on diverse patient populations and long-term outcomes.

- The phase III study NCT05063149 includes 500 moderate–late preterm infants aged 6 to 10 weeks who will receive OM-85 until 12 months after birth. This study evaluates the efficacy of OM-85 in the reduction in RTIs and wheezing in the first years of life. This study will also contribute to determining the correlation of biological markers with respiratory symptoms, immune protection and treatment effect [120].
- The multicenter phase II study NCT05857930 assesses the efficacy and safety of daily OM-85 treatment as an adjunct to standard care versus placebo in reducing wheezing/asthma-like episodes (WEs) over a 6-month period in children aged 6 months to 5 years with a history of recurrent WEs [122].
- The ORBEX trial (NCT02148796) evaluates whether OM-85 can increase time to first wheezing lower respiratory tract illness (WLRI) episode in high-risk infants. The study enrolled 822 children aged 6–18 months who received treatment for 2 consecutive years, followed by a 3-year observational phase off therapy. This large-cohort study will provide valuable long-term efficacy data in a high-risk population [123].

*Lactobacillus rhamnosus* CRL1505: First Dedicated Pediatric RTI Trial: The NCT07154992 trial is a randomized, double-blind, placebo-controlled study in 268 healthy children aged 3–12 years, assessing whether daily intake of CRL1505 for 12 weeks, with a 4-week follow-up, can reduce the incidence, severity, and duration of URTIs [124]. This trial is expected to provide the first large-scale validation of CRL1505 as a preventive immunobiotic in pediatrics and will determine whether the substantial mechanistic data translate into clinical efficacy.

Research Priorities for PMBL and MV130: By contrast, no ongoing pediatric RCTs were identified for MV130 (Bactek/Bacmune) or PMBL (Ismigen) as of September 2025 on ClinicalTrials.gov. This represents a critical research gap, particularly for MV130, which has shown substantial efficacy in its single RCT but requires replication and expansion across broader populations and clinical phenotypes. The absence of ongoing trials for PMBL similarly limits the ability to establish definitive efficacy beyond the preliminary meta-analytic evidence.

## 6. Discussion

This narrative review reveals substantial heterogeneity in clinical evidence maturity across four bacterial-derived immunomodulators for pediatric RTI prevention, with direct implications for evidence-based practice.

Across the agents reviewed, OM-85 has the most consistent clinical evidence and may be considered in selected pediatric patients (e.g., recurrent RTIs), while recognizing variability in effect estimates, endpoints, and methodological quality across trials. In 18 pediatric RCTs and seven meta-analyses, it has shown consistent 26–36% RTI reductions and benefits for wheezing/asthma exacerbations [87,88,89,90,91,92,93]. PMBL and MV130 demonstrated preliminary efficacy, reducing infectious episodes by a mean of 2.20 (*p* < 0.0001) [19] and 40% [20], respectively, but require larger trials for definitive recommendations. While CRL1505 lacks dedicated pediatric RTI trials, ongoing studies will determine whether its preclinical promise translates into clinical efficacy [76,86]. Despite this evidence of heterogeneity, all four agents share core mechanisms (epithelial strengthening, innate and adaptive immunity activation and inflammation control), with specific research focus for each product. While MV130’s trained immunity induction [108] and CRL1505’s gut–lung axis modulation [73,86] have been documented, these findings warrant further comparative investigation. From a clinical standpoint, these findings are most directly relevant to practicing pediatricians managing children with recurrent respiratory infections—particularly those experiencing three or more RTI episodes per year, those with recurrent virus-induced wheezing, or those in high-exposure settings such as daycare [87,90]. It is important to emphasize that none of the products reviewed here can replace standard preventive measures, including vaccination, breastfeeding promotion, and reduction in environmental risk factors; rather, bacterial-derived immunomodulators may be considered as a complementary strategy in selected clinical scenarios.

A critical challenge limiting clinical adoption is the substantial heterogeneity in study designs and outcomes across trials. As Castro-Rodriguez et al. have highlighted, clinical trials of bacterial-derived immunomodulators exhibit wide variability in populations, dosing schedules, comparators, and outcome definitions [125], reflected in meta-analyses demonstrating considerable inter-study variability and suboptimal methodological quality [87,92]. The standardization of trial protocols (including harmonized core endpoints, age-stratified analyses, and prospective registration with transparent reporting) is crucial to improve evidence quality and enable meaningful cross-study comparisons [125].

Additionally, most clinical trials have focused on short-term outcomes, such as reductions in RTIs or wheezing over 3–12 months [90,95,111], leaving important gaps in understanding long-term effects on immune maturation and respiratory health trajectories. As a result, the certainty of evidence regarding long-term preventive effects remains limited, precluding high-certainty or strong recommendations for routine use in asthma prevention. Addressing this gap, the ORBEX trial (NCT02148796), with its 5-year follow-up, will be pivotal in determining whether early immunomodulation can delay or prevent the first episode of wheezing lower respiratory tract illness [123].

Incomplete mechanistic characterization currently limits optimization and personalization of bacterial-derived immunomodulator therapy. Although these agents share convergent pathways, the molecular determinants of their clinical effects remain only partially defined. Large-scale immunophenotyping studies have shown that innate and adaptive immune traits are differentially shaped by genetic and environmental factors [126,127], likely contributing to inter-individual variability in immunomodulator efficacy. To address such mechanistic uncertainties, future research should integrate systems-immunology approaches to identify predictive biomarkers (baseline IFN signatures, microbiome profiles) and define responder phenotypes, enabling precision immunomodulation.

Safety is paramount in pediatric populations. OM-85 has generally shown favorable safety profiles in pediatric RCTs, with no increase in serious adverse events, and good tolerability even when co-administered with the influenza vaccination [89,95,101]. Nevertheless, children with primary immunodeficiencies, severe atopic predisposition, or chronic comorbidities may respond unpredictably [128]. Emerging data from ongoing OM-85 trials in high-risk populations represent initial steps toward addressing these gaps [121,123]. However, substantial additional research is needed to generate comparable evidence for other products. Safety monitoring in ongoing and future pediatric trials remains essential to ensure benefit–risk consistency across broader populations.

Implementation barriers include inconsistent regulatory pathways across regions and product classifications [30,128,129], complex dosing schedules, and cost/accessibility considerations [130,131,132], all of which may affect adherence and access. Strategies to address these barriers include simplified dosing schedules, reimbursement frameworks, and educational programs for healthcare providers and families. Overcoming these barriers will be key to translating the immunobiotic concept into real-world pediatric prevention strategies.

This review provides the first comprehensive synthesis of preclinical mechanisms and clinical evidence across four distinct bacterial-derived immunomodulators, spanning the full spectrum of evidence maturity: from extensively preclinical and clinical data (OM-85) [87,90,93,133] to extensive preclinical evidence with limited clinical validation (PMBL, MV130) [19,20,66,134] to robust preclinical data with ongoing first-in-human evaluation in pediatric populations (CRL1505) [76,78,79,124]. The parallel presentation of mechanistic data and clinical outcomes enables an assessment of biological plausibility alongside evidence robustness.

As a narrative review, our synthesis was not based on formal systematic review methodology with prospectively registered protocols. We restricted searches to PubMed, potentially missing studies indexed exclusively in other databases, though PubMed provides comprehensive coverage of the pediatric and immunology literature. Our focus on four specific products, while enabling depth of analysis, excluded other bacterial lysates and probiotics that may have relevant evidence. Given the rapid evolution of this field, ongoing and future studies may substantially reshape the evidence hierarchy after the publication of this review. In addition, it is important to highlight that children with recurrent respiratory symptoms, persistent wheeze, atypical clinical features, or poor response to preventive strategies should undergo careful clinical reassessment and appropriate evaluation for alternative or coexisting conditions, including structural or anatomical airway abnormalities, aspiration syndrome, cystic fibrosis, primary ciliary dyskinesia, immunodeficiencies, bronchiectasis, and others when clinically indicated.

## 7. Conclusions

The strength of clinical evidence varies across products. OM-85’s clinical benefit is supported by 18 pediatric randomized controlled trials and seven meta-analyses, consistently demonstrating reductions in RTI frequency and wheezing or asthma exacerbations. PMBL and MV130 present encouraging preliminary data requiring validation in large multicenter trials, while *Lactobacillus rhamnosus* CRL1505 presents strong mechanistic plausibility awaiting clinical confirmation.

The early-childhood immune window represents a crucial opportunity for preventive interventions to interrupt the RTI–asthma continuum. Through the restoration of immune homeostasis, bacterial-derived immunomodulators could help reshape respiratory health trajectories in children. Although the maturity of evidence differs among products, the convergence of mechanistic insight, clinical efficacy, and favorable safety profiles supports their consideration as emerging components of evidence-based preventive strategies.

Ongoing large-scale studies will be instrumental in closing evidence gaps and determining their place in future pediatric respiratory care guidelines.

## Figures and Tables

**Table 1:** Clinical evidence for pediatric bacterial-derived immunomodulators from randomized controlled trials and meta-analyses.

Products	Numbers of RCT	Total Number of Patients in RCT	Numbers of Meta-Analyses	Refs
Polyvalent chemical lysate (OM-85)	18	2116	7	[87,88,89,90,91,92,93,94,95,96,97,98,99,100,101,102,103,104,105,106,107,108,109]
PMBL (Ismigen)	5	420	1	[19,110,111,112,113,114]
Heat-inactivated whole bacteria (MV130)	1	120	0	[102]
Probiotic (CRL1505)	0	0	0	

For each product, the number of RCTs and meta-analyses identified through structured PubMed search (September 2025) is presented. RCT columns indicate the number of RCTs for each product, along with the total number of patients enrolled. Meta-analysis columns indicate the number of meta-analyses identified for each product. Complete study characteristics are provided in Supplementary Materials S2: Tables S5–S7. Abbreviations: RCT, randomized controlled trial; PMBL, polyvalent mechanical bacterial lysate; Refs, references.

**Table 2:** Ongoing pediatric trials of OM-85, PMBL, MV130, and CRL1505.

Products	Trial ID	Phase	N	Population	Primary Endpoint	Duration	Status	Refs
OM-85	NCT05677763	IV	525	Children aged 6 months to 5 years with multiple RTIs	Rate of RTIs	12 months, and 6 months of observation	Active, not recruiting	[119]
OM-85	NCT05063149	III	500	Preterm children aged 6 to 10 weeks	Total LRTIs and wheezing episodes in first years of life	12 months after birth	Recruiting	[120]
OM-85	NCT05064631 (BLIPA)	IIb	173	Children aged 3 to 12 months with severe bronchiolitis	Presence of wheezing	19 and 24 months	Active, not recruiting	[121]
OM-85	NCT05857930	II	288	Children aged 6 months to 5 years with recurrent wheezing	Rate of wheezing/asthma-like episodes	6 months	Active, not recruiting	[122]
OM-85	NCT02148796 (ORBEX)	II	822	Children aged 6 to 18 months at risk of wheezing and asthma	Time to the first WLRI episode in the observation period after two consecutive years of therapy	5 years	Completed	[123]
CRCL1505	NCT07154992	NA	268	Healthy children aged 3 to 12 years	Reduction or prevention URTIs	12 weeks and 4 additional weeks post-treatment	Recruiting	[124]
PBML	No registered pediatric trials identified							
MV130	No registered pediatric trials identified							

Trials include ongoing pediatric studies (recruiting, active, not recruiting) identified on ClinicalTrials.gov. Columns report product, registry identifier, phase, population, primary endpoint, the duration and the completion of the studies, and status. Pediatric eligibility was verified from registry age fields and clinical context. NA denotes “not applicable”; trial phase designation does not apply to probiotic products CRL1505. No registered pediatric trials identified for PMBL (Ismigen) or MV130 on ClinicalTrials.gov at the time of the search. Data were extracted on 15 September 2025; registry records are dynamic and may be updated after this date. Abbreviations: Refs, references; LRTI, lower respiratory tract infection; PMBL, polyvalent mechanical bacterial lysate; RTI, respiratory tract infection; URTI, upper respiratory tract infection; WLRI, wheeze-like respiratory illness; NA, not applicable.

## Data Availability

No new data were created or analyzed in this study. Data sharing is not applicable to this article.

## Supplementary Materials

The following supporting information can be downloaded at: https://www.mdpi.com/article/10.3390/children13060737/s1, Supplementary Materials S1: Search strategies and methodology; Supplementary Materials S2 (Tables S1–S7): Characteristics of included preclinical and clinical studies.

## Abbreviations

The following abbreviations are used in this manuscript:

ACE2	Angiotensin-converting enzyme 2
ALRI	Acute lower respiratory infection
CCL	Chemokine (C-C motif) ligand
CD4	Cluster of differentiation 4
CD8	Cluster of differentiation 8
CFU	Colony-forming units
COPD	Chronic obstructive pulmonary disease
CRL1505	*Lactobacillus rhamnosus* CRL1505
CXCL	Chemokine (C-X-C motif) ligand
DC	Dendritic cell
G-CSF	Granulocyte colony-stimulating factor
hMPV	Human metapneumovirus
hPIV	Human parainfluenza virus
HR	Hazard ratio
ICAM-1	Intercellular adhesion molecule 1
IFN-β	Interferon beta
IFN-γ	Interferon gamma
IFV	Influenza virus
IgA	Immunoglobulin A
IgE	Immunoglobulin E
IgG	Immunoglobulin G
IL	Interleukin
ILC2	Group-2 innate lymphoid cells
ILC3	Type 3 innate lymphoid cells
IQR	Interquartile range
LRTI	Lower respiratory tract infection
MAPK	Mitogen-activated protein kinases
MD	Mean difference
MHC-II	Major histocompatibility complex class II
mTORC1	Mammalian target of rapamycin complex 1
MV130	Mucosal vaccine 130
MyD88	Myeloid differentiation primary response 88
NF-κB	Nuclear factor kappa B
NK	Natural killer
NO	Nitric oxide
OXPHOS	Oxidative phosphorylation
PG	Peptidoglycan
PMBL	Polyvalent mechanical bacterial lysate
PRR	Pattern recognition receptor
RCT	Randomized controlled trial
RIG-I	Retinoic acid-inducible gene I
RIPK2	Receptor-interacting protein kinase 2
RR	Relative risk
RSV	Respiratory syncytial virus
RTI	Respiratory tract infection
RV	Rhinovirus
SARS-CoV-2	Severe acute respiratory syndrome coronavirus 2
SCFA	Short-chain fatty acids
TBK1	TANK-binding kinase 1
TEER	Transepithelial resistance
Th1	T helper type 1
Th2	T helper type 2
Th17	T helper type 17
TLR	Toll-like receptor
TMPRSS2	Transmembrane serine protease 2
TNF-α	Tumor necrosis factor alpha
Treg	Regulatory T cell
TSLP	Thymic stromal lymphopoietin
URTI	Upper respiratory tract infections
WLRI	Wheezing lower respiratory tract illness
ZO-1I	Zonula occludens-1

## References

[1] He L., Weng J., Zhu F., Zhang Y., Chen J., Chen S., Lu M., Nair H., Li Y., Wang X. (2025). The Morbidity Spectrum of Influenza, Respiratory Syncytial Virus, Human Metapneumovirus and Human Parainfluenza Virus in Young Children by Age and Country Income Level: A Systematic Review and Meta-Analysis. Int. J. Infect. Dis..

[2] Vandini S., Biagi C., Fischer M., Lanari M. (2019). Impact of Rhinovirus Infections in Children. Viruses.

[3] Suleiman-Martos N., Caballero-Vázquez A., Gómez-Urquiza J.L., Albendín-García L., Romero-Béjar J.L., Cañadas-De La Fuente G.A. (2021). Prevalence and Risk Factors of Respiratory Syncytial Virus in Children under 5 Years of Age in the WHO European Region: A Systematic Review and Meta-Analysis. J. Pers. Med..

[4] Li Y., Wang X., Blau D.M., Caballero M.T., Feikin D.R., Gill C.J., Madhi S.A., Omer S.B., Simões E.A.F., Campbell H. (2022). Global, Regional, and National Disease Burden Estimates of Acute Lower Respiratory Infections Due to Respiratory Syncytial Virus in Children Younger than 5 Years in 2019: A Systematic Analysis. Lancet.

[5] McLaughlin J.M., Khan F., Schmitt H.-J., Agosti Y., Jodar L., Simões E.A.F., Swerdlow D.L. (2022). Respiratory Syncytial Virus–Associated Hospitalization Rates among US Infants: A Systematic Review and Meta-Analysis. J. Infect. Dis..

[6] Del Riccio M., Spreeuwenberg P., Osei-Yeboah R., Johannesen C.K., Fernandez L.V., Teirlinck A.C., Wang X., Heikkinen T., Bangert M., Caini S. (2023). Burden of Respiratory Syncytial Virus in the European Union: Estimation of RSV-Associated Hospitalizations in Children under 5 Years. J. Infect. Dis..

[7] Yu J.C., Khodadadi H., Malik A., Davidson B., da Silva Lopes Salles É., Bhatia J., Hale V.L., Baban B. (2018). Innate Immunity of Neonates and Infants. Front. Immunol..

[8] Moraes-Pinto M.I.D., Suano-Souza F., Aranda C.S. (2021). Immune System: Development and Acquisition of Immunological Competence. J. Pediatr..

[9] Pieren D.K.J., Boer M.C., De Wit J. (2022). The Adaptive Immune System in Early Life: The Shift Makes It Count. Front. Immunol..

[10] Goenka A., Kollmann T.R. (2015). Development of Immunity in Early Life. J. Infect..

[11] Tregoning J.S., Schwarze J. (2010). Respiratory Viral Infections in Infants: Causes, Clinical Symptoms, Virology, and Immunology. Clin. Microbiol. Rev..

[12] Edwards M.R., Walton R.P., Jackson D.J., Feleszko W., Skevaki C., Jartti T., Makrinoti H., Nikonova A., Shilovskiy I.P., Schwarze J. (2018). The Potential of Anti-infectives and Immunomodulators as Therapies for Asthma and Asthma Exacerbations. Allergy.

[13] Kloepfer K.M., Jackson D.J., Jartti T., Liu A.H., Gern J.E. (2025). How Infections and Immune Development Relate to Preschool Recurrent Wheezing and Asthma. J. Allergy Clin. Immunol. Pract..

[14] Mikhail I., Grayson M.H. (2019). Asthma and Viral Infections. Ann. Allergy Asthma Immunol..

[15] Liu L., Pan Y., Zhu Y., Song Y., Su X., Yang L., Li M. (2017). Association between Rhinovirus Wheezing Illness and the Development of Childhood Asthma: A Meta-Analysis. BMJ Open.

[16] Ma R., Zhang C., Zhang Y., Tan H., Zhang Y., Li Q., Bai Y., Sun X. (2025). The Impact of Respiratory Syncytial Virus on Asthma Development and Exacerbation. Ann. Allergy Asthma Immunol..

[17] Dang A.T., Pasquali C., Ludigs K., Guarda G. (2017). OM-85 Is an Immunomodulator of Interferon-β Production and Inflammasome Activity. Sci. Rep..

[18] Troy N.M., Strickland D., Serralha M., De Jong E., Jones A.C., Read J., Galbraith S., Islam Z., Kaur P., Mincham K.T. (2022). Protection against Severe Infant Lower Respiratory Tract Infections by Immune Training: Mechanistic Studies. J. Allergy Clin. Immunol..

[19] Cazzola M., Anapurapu S., Page C.P. (2012). Polyvalent Mechanical Bacterial Lysate for the Prevention of Recurrent Respiratory Infections: A Meta-Analysis. Pulm. Pharmacol. Ther..

[20] Nieto A., Mazón A., Nieto M., Calderón R., Calaforra S., Selva B., Uixera S., Palao M.J., Brandi P., Conejero L. (2021). Bacterial Mucosal Immunotherapy with MV130 Prevents Recurrent Wheezing in Children: A Randomized, Double-Blind, Placebo-Controlled Clinical Trial. Am. J. Respir. Crit. Care Med..

[21] Montalbán-Hernández K., Cogollo-García A., Girón De Velasco-Sada P., Caballero R., Casanovas M., Subiza J.L., Conejero L. (2024). MV130 in the Prevention of Recurrent Respiratory Tract Infections: A Retrospective Real-World Study in Children and Adults. Vaccines.

[22] Du T., Lei A., Zhang N., Zhu C. (2022). The Beneficial Role of Probiotic Lactobacillus in Respiratory Diseases. Front. Immunol..

[23] Kenmoe S., Atenguena Okobalemba E., Takuissu G.R., Ebogo-Belobo J.T., Oyono M.G., Magoudjou-Pekam J.N., Kame-Ngasse G.I., Taya-Fokou J.B., Mbongue Mikangue C.A., Kenfack-Momo R. (2022). Association between Early Viral Lower Respiratory Tract Infections and Subsequent Asthma Development. World J. Crit. Care Med..

[24] Binns E., Tuckerman J., Licciardi P.V., Wurzel D. (2022). Respiratory Syncytial Virus, Recurrent Wheeze and Asthma: A Narrative Review of Pathophysiology, Prevention and Future Directions. J. Paediatr. Child Health.

[25] Le Souëf P. (2018). Viral Infections in Wheezing Disorders. Eur. Respir. Rev..

[26] Kombe Kombe A.J., Fotoohabadi L., Gerasimova Y., Nanduri R., Lama Tamang P., Kandala M., Kelesidis T. (2024). The Role of Inflammation in the Pathogenesis of Viral Respiratory Infections. Microorganisms.

[27] Mthembu N., Ikwegbue P., Brombacher F., Hadebe S. (2021). Respiratory Viral and Bacterial Factors That Influence Early Childhood Asthma. Front. Allergy.

[28] Luo W., Hu J., Xu W., Dong J. (2022). Distinct Spatial and Temporal Roles for Th1, Th2, and Th17 Cells in Asthma. Front. Immunol..

[29] Semmes E.C., Chen J.-L., Goswami R., Burt T.D., Permar S.R., Fouda G.G. (2021). Understanding Early-Life Adaptive Immunity to Guide Interventions for Pediatric Health. Front. Immunol..

[30] Di Gioacchino M., Santilli F., Pession A. (2024). Is There a Role for Immunostimulant Bacterial Lysates in the Management of Respiratory Tract Infection?. Biomolecules.

[31] Mastrangelo P., Chin A.A., Tan S., Jeon A.H., Ackerley C.A., Siu K.K., Lee J.E., Hegele R.G. (2021). Identification of RSV Fusion Protein Interaction Domains on the Virus Receptor, Nucleolin. Viruses.

[32] Ganjian H., Rajput C., Elzoheiry M., Sajjan U. (2020). Rhinovirus and Innate Immune Function of Airway Epithelium. Front. Cell. Infect. Microbiol..

[33] Pace E., Di Vincenzo S., Ferraro M., Lanata L., Scaglione F. (2024). Role of Airway Epithelium in Viral Respiratory Infections: Can Carbocysteine Prevent or Mitigate Them?. Immunology.

[34] Roan F., Obata-Ninomiya K., Ziegler S.F. (2019). Epithelial Cell–Derived Cytokines: More than Just Signaling the Alarm. J. Clin. Investig..

[35] Sasaki H., Miyata J., Kawana A., Fukunaga K. (2025). Antiviral Roles of Eosinophils in Asthma and Respiratory Viral Infection. Front. Allergy.

[36] Malinczak C.-A., Fonseca W., Hrycaj S.M., Morris S.B., Rasky A.J., Yagi K., Wellik D.M., Ziegler S.F., Zemans R.L., Lukacs N.W. (2024). Early-Life Pulmonary Viral Infection Leads to Long-Term Functional and Lower Airway Structural Changes in the Lungs. Am. J. Physiol. Lung Cell. Mol. Physiol..

[37] Rosas-Salazar C., Chirkova T., Gebretsadik T., Chappell J.D., Peebles R.S., Dupont W.D., Jadhao S.J., Gergen P.J., Anderson L.J., Hartert T.V. (2023). Respiratory Syncytial Virus Infection during Infancy and Asthma during Childhood in the USA (INSPIRE): A Population-Based, Prospective Birth Cohort Study. Lancet.

[38] Rossi G.A., Pohunek P., Feleszko W., Ballarini S., Colin A.A. (2020). Viral Infections and Wheezing–Asthma Inception in Childhood: Is There a Role for Immunomodulation by Oral Bacterial Lysates?. Clin. Transl. Allergy.

[39] Beigelman A., Bacharier L.B. (2016). Early-Life Respiratory Infections and Asthma Development: Role in Disease Pathogenesis and Potential Targets for Disease Prevention. Curr. Opin. Allergy Clin. Immunol..

[40] Achten N.B., Van Rossum A.M.C., Bacharier L.B., Fitzpatrick A.M., Hartert T.V. (2022). Long-Term Respiratory Consequences of Early-Life Respiratory Viral Infections: A Pragmatic Approach to Fundamental Questions. J. Allergy Clin. Immunol. Pract..

[41] Kaczynska A., Klosinska M., Janeczek K., Zarobkiewicz M., Emeryk A. (2022). Promising Immunomodulatory Effects of Bacterial Lysates in Allergic Diseases. Front. Immunol..

[42] Loisel D.A., Du G., Ahluwalia T.S., Tisler C.J., Evans M.D., Myers R.A., Gangnon R.E., Kreiner-Møller E., Bønnelykke K., Bisgaard H. (2016). Genetic Associations with Viral Respiratory Illnesses and Asthma Control in Children. Clin. Exp. Allergy.

[43] Castro-Rodriguez J.A., Wolters A.A.B., Rodriguez-Martinez C.E., Biagini J.M., Celedón J.C., Custovic A., Koppelman G.H., Phipatanakal W., Saglani S., Forno E. (2025). Risk Factors and Mechanisms Leading to Preschool Recurrent Wheeze and Asthma. J. Allergy Clin. Immunol. Pract..

[44] Koppelman G.H., Pino-Yanes M., Melén E., Powell P., Bracke K.R., Celedón J.C., Brusselle G.G. (2025). Genetic and Environmental Risk Factors for Asthma: Towards Prevention. Lancet Respir. Med..

[45] De Benedetto F., Sevieri G. (2013). Prevention of Respiratory Tract Infections with Bacterial Lysate OM-85 Bronchomunal in Children and Adults: A State of the Art. Multidiscip. Respir. Med..

[46] Kearney S.C., Dziekiewicz M., Feleszko W. (2015). Immunoregulatory and Immunostimulatory Responses of Bacterial Lysates in Respiratory Infections and Asthma. Ann. Allergy Asthma Immunol..

[47] Morandi B., Agazzi A., D’Agostino A., Antonini F., Costa G., Sabatini F., Ferlazzo G., Melioli G. (2011). A Mixture of Bacterial Mechanical Lysates Is More Efficient than Single Strain Lysate and of Bacterial-Derived Soluble Products for the Induction of an Activating Phenotype in Human Dendritic Cells. Immunol. Lett..

[48] Brandi P., Conejero L., Cueto F.J., Martínez-Cano S., Dunphy G., Gómez M.J., Relaño C., Saz-Leal P., Enamorado M., Quintas A. (2022). Trained Immunity Induction by the Inactivated Mucosal Vaccine MV130 Protects against Experimental Viral Respiratory Infections. Cell Rep..

[49] Ozen M., Kocabas Sandal G., Dinleyici E.C. (2015). Probiotics for the Prevention of Pediatric Upper Respiratory Tract Infections: A Systematic Review. Expert Opin. Biol. Ther..

[50] Roth M., Pasquali C., Stolz D., Tamm M. (2017). Broncho Vaxom (OM-85) Modulates Rhinovirus Docking Proteins on Human Airway Epithelial Cells via Erk1/2 Mitogen Activated Protein Kinase and cAMP. PLoS ONE.

[51] Roth M., Khameneh H.J., Fang L., Tamm M., Rossi G.A. (2021). Distinct Antiviral Properties of Two Different Bacterial Lysates. Can. Respir. J..

[52] Triantafillou V., Workman A.D., Patel N.N., Maina I.W., Tong C.C.L., Kuan E.C., Kennedy D.W., Palmer J.N., Adappa N.D., Waizel-Haiat S. (2019). Broncho-Vaxom^®^ (OM-85 BV) Soluble Components Stimulate Sinonasal Innate Immunity. Int. Forum Allergy Rhinol..

[53] Pivniouk V., Gimenes-Junior J.A., Ezeh P., Michael A., Pivniouk O., Hahn S., VanLinden S.R., Malone S.P., Abidov A., Anderson D. (2022). Airway Administration of OM-85, a Bacterial Lysate, Blocks Experimental Asthma by Targeting Dendritic Cells and the Epithelium/IL-33/ILC2 Axis. J. Allergy Clin. Immunol..

[54] Navarro S., Cossalter G., Chiavaroli C., Kanda A., Fleury S., Lazzari A., Cazareth J., Sparwasser T., Dombrowicz D., Glaichenhaus N. (2011). The Oral Administration of Bacterial Extracts Prevents Asthma via the Recruitment of Regulatory T Cells to the Airways. Mucosal Immunol..

[55] Fu R., Li J., Zhong H., Yu D., Zeng X., Deng M., Sun Y., Wen W., Li H. (2014). Broncho-Vaxom Attenuates Allergic Airway Inflammation by Restoring GSK3β-Related T Regulatory Cell Insufficiency. PLoS ONE.

[56] Huber M., Mossmann H., Bessler W.G. (2005). Th1-orientated immunological properties of the bacterial extract OM-85-BV. Eur. J. Med. Res..

[57] Khameneh H.J., Bolis M., Ventura P.M.O., Cassanmagnago G.A., Fischer B.A., Zenobi A., Guerra J., Buzzago I., Bernasconi M., Zaman G.J.R. (2024). The Bacterial Lysate OM-85 Engages Toll-like Receptors 2 and 4 Triggering an Immunomodulatory Gene Signature in Human Myeloid Cells. Mucosal Immunol..

[58] Parola C., Salogni L., Vaira X., Scutera S., Somma P., Salvi V., Musso T., Tabbia G., Bardessono M., Pasquali C. (2013). Selective Activation of Human Dendritic Cells by OM-85 through a NF-kB and MAPK Dependent Pathway. PLoS ONE.

[59] Zelle-Rieser C., Ramoner R., Bartsch G., Thurnher M. (2001). A Clinically Approved Oral Vaccine against Pneumotropic Bacteria Induces the Terminal Maturation of CD83+ Immunostimulatory Dendritic Cells. Immunol. Lett..

[60] Pasquali C., Salami O., Taneja M., Gollwitzer E.S., Trompette A., Pattaroni C., Yadava K., Bauer J., Marsland B.J. (2014). Enhanced Mucosal Antibody Production and Protection against Respiratory Infections Following an Orally Administered Bacterial Extract. Front. Med..

[61] Antunes K.H., Cassão G., Santos L.D., Borges S.G., Poppe J., Gonçalves J.B., Nunes E.D.S., Recacho G.F., Sousa V.B., Da Silva G.S. (2022). Airway Administration of Bacterial Lysate OM-85 Protects Mice Against Respiratory Syncytial Virus Infection. Front. Immunol..

[62] Rossi G.A., Bessler W., Ballarini S., Pasquali C. (2018). Evidence That a Primary Anti-Viral Stimulation of the Immune Response by OM-85 Reduces Susceptibility to a Secondary Respiratory Bacterial Infection in Mice. Ital. J. Pediatr..

[63] Byl B., Libin M., Gérard M., Clumeck N., Goldman M., Mascart-Lemone F. (1998). Bacterial Extract OM85-BV Induces Interleukin-12-Dependent IFN-γ Production by Human CD4+ T Cells. J. Interferon Cytokine Res..

[64] Sidoti Migliore G., Campana S., Barberi C., De Pasquale C., Pezzino G., Cavaliere R., Orecchia P., Ginestra G., Mandalari G., Del Zotto G. (2023). Mechanical Bacterial Lysate Enhances Antimicrobial Barrier Mechanisms in Human Airway Epithelial Cells. J. Leukoc. Biol..

[65] Ferrara F., Rial A., Suárez N., Chabalgoity J.A. (2021). Polyvalent Bacterial Lysate Protects Against Pneumonia Independently of Neutrophils, IL-17A or Caspase-1 Activation. Front. Immunol..

[66] Braido F., Melioli G., Nicolini G., Ferraris M., Di Girolamo S., Di Gioacchino M., Canonica G.W. (2024). Sublingually Administered Bacterial Lysates: Rationale, Mechanisms of Action and Clinical Outcomes. Drugs Context.

[67] Lanzilli G., Falchetti R., Tricarico M., Ungheri D., Fuggetta M.P. (2005). In Vitro Effects of an Immunostimulating Bacterial Lysate on Human Lymphocyte Function. Int. J. Immunopathol. Pharmacol..

[68] Lanzilli G., Falchetti R., Cottarelli A., Macchi A., Ungheri D., Fuggetta M.P. (2006). In Vivo Effect of an Immunostimulating Bacterial Lysate on Human B Lymphocytes. Int. J. Immunopathol. Pharmacol..

[69] Sevilla-Ortega C., Angelina A., Martín-Cruz L., Pérez-Diego M., Maldonado A., Lavín B., Marcos-Ramiro B., Pérez De Llano L., Gayá A., Real F.X. (2025). A Mucosal Vaccine Prevents Eosinophilic Allergic Airway Inflammation by Modulating Immune Responses to Allergens in a Murine Model of Airway Disease. Nat. Commun..

[70] Cirauqui C., Benito-Villalvilla C., Sánchez-Ramón S., Sirvent S., Diez-Rivero C.M., Conejero L., Brandi P., Hernández-Cillero L., Ochoa J.L., Pérez-Villamil B. (2018). Human Dendritic Cells Activated with MV130 Induce Th1, Th17 and IL-10 Responses via RIPK2 and MyD88 Signalling Pathways. Eur. J. Immunol..

[71] Del Fresno C., García-Arriaza J., Martínez-Cano S., Heras-Murillo I., Jarit-Cabanillas A., Amores-Iniesta J., Brandi P., Dunphy G., Suay-Corredera C., Pricolo M.R. (2021). The Bacterial Mucosal Immunotherapy MV130 Protects Against SARS-CoV-2 Infection and Improves COVID-19 Vaccines Immunogenicity. Front. Immunol..

[72] Vázquez A., Fernández-Sevilla L.M., Jiménez E., Pérez-Cabrera D., Yañez R., Subiza J.L., Varas A., Valencia J., Vicente A. (2020). Involvement of Mesenchymal Stem Cells in Oral Mucosal Bacterial Immunotherapy. Front. Immunol..

[73] Albarracin L., Garcia-Castillo V., Masumizu Y., Indo Y., Islam M.A., Suda Y., Garcia-Cancino A., Aso H., Takahashi H., Kitazawa H. (2020). Efficient Selection of New Immunobiotic Strains with Antiviral Effects in Local and Distal Mucosal Sites by Using Porcine Intestinal Epitheliocytes. Front. Immunol..

[74] Zhou B., Elean M., Arce L., Fukuyama K., Tomotsune K., Dentice Maidana S., Saha S., Namai F., Nishiyama K., Vizoso-Pinto M.G. (2024). The Mucus-Binding Factor Mediates *Lacticaseibacillus rhamnosus* CRL1505 Adhesion but Not Immunomodulation in the Respiratory Tract. Microorganisms.

[75] Dentice Maidana S., Imamura Y., Elean M., Albarracín L., Nishiyama K., Suda Y., Kurata S., Jure M.Á., Kitazawa H., Villena J. (2023). Oral Administration of *Lacticaseibacillus rhamnosus* CRL1505 Modulates Lung Innate Immune Response against Klebsiella Pneumoniae ST25. Microorganisms.

[76] Tomotsune K., Raya Tonetti F., Mizuno H., Elean M., Fukuyama K., Zhou B., Ikeda-Ohtsubo W., Nishiyama K., Yamamura A., Karasawa H. (2022). The Mucus Binding Factor Is Not Necessary for *Lacticaseibacillus rhamnosus* CRL1505 to Exert Its Immunomodulatory Activities in Local and Distal Mucosal Sites. Int. J. Mol. Sci..

[77] Clua P., Kanmani P., Zelaya H., Tada A., Kober A.K.M.H., Salva S., Alvarez S., Kitazawa H., Villena J. (2017). Peptidoglycan from Immunobiotic *Lactobacillus rhamnosus* Improves Resistance of Infant Mice to Respiratory Syncytial Viral Infection and Secondary Pneumococcal Pneumonia. Front. Immunol..

[78] Clua P., Tomokiyo M., Raya Tonetti F., Islam M.A., García Castillo V., Marcial G., Salva S., Alvarez S., Takahashi H., Kurata S. (2020). The Role of Alveolar Macrophages in the Improved Protection against Respiratory Syncytial Virus and Pneumococcal Superinfection Induced by the Peptidoglycan of *Lactobacillus rhamnosus* CRL1505. Cells.

[79] Raya Tonetti F., Clua P., Fukuyama K., Marcial G., Sacur J., Marranzino G., Tomokiyo M., Vizoso-Pinto G., Garcia-Cancino A., Kurata S. (2022). The Ability of Postimmunobiotics from L. Rhamnosus CRL1505 to Protect against Respiratory Syncytial Virus and Pneumococcal Super-Infection Is a Strain-Dependent Characteristic. Microorganisms.

[80] Esposito S., Jones M.H., Feleszko W., Martell J.A.O., Falup-Pecurariu O., Geppe N., Martinón-Torres F., Shen K.-L., Roth M., Principi N. (2020). Prevention of New Respiratory Episodes in Children with Recurrent Respiratory Infections: An Expert Consensus Statement from the World Association of Infectious Diseases and Immunological Disorders (WAidid). Microorganisms.

[81] Pivniouk V., Pivniouk O., DeVries A., Uhrlaub J.L., Michael A., Pivniouk D., VanLinden S.R., Conway M.Y., Hahn S., Malone S.P. (2022). The OM-85 Bacterial Lysate Inhibits SARS-CoV-2 Infection of Epithelial Cells by Downregulating SARS-CoV-2 Receptor Expression. J. Allergy Clin. Immunol..

[82] Fang L., Zhou L., Tamm M., Roth M. (2021). OM-85 Broncho-Vaxom^®^, a Bacterial Lysate, Reduces SARS-CoV-2 Binding Proteins on Human Bronchial Epithelial Cells. Biomedicines.

[83] Bessler W.G., Vor Dem Esche U., Masihi N. (2010). The Bacterial Extract OM-85 BV Protects Mice against Influenza and Salmonella Infection. Int. Immunopharmacol..

[84] Banche G., Allizond V., Mandras N., Garzaro M., Cavallo G.P., Baldi C., Scutera S., Musso T., Roana J., Tullio V. (2007). Improvement of Clinical Response in Allergic Rhinitis Patients Treated with an Oral Immunostimulating Bacterial Lysate: In Vivo Immunological Effects. Int. J. Immunopathol. Pharmacol..

[85] Villena J., Chiba E., Vizoso-Pinto M., Tomosada Y., Takahashi T., Ishizuka T., Aso H., Salva S., Alvarez S., Kitazawa H. (2014). Immunobiotic *Lactobacillus rhamnosus* Strains Differentially Modulate Antiviral Immune Response in Porcine Intestinal Epithelial and Antigen Presenting Cells. BMC Microbiol..

[86] Garcia-Castillo V., Tomokiyo M., Raya Tonetti F., Islam M.A., Takahashi H., Kitazawa H., Villena J. (2020). Alveolar Macrophages Are Key Players in the Modulation of the Respiratory Antiviral Immunity Induced by Orally Administered *Lacticaseibacillus rhamnosus* CRL1505. Front. Immunol..

[87] Berber A., Del-Río-Navarro B.E., Reyes-Noriega N., Sienra-Monge J.J.L. (2022). Immunostimulants for Preventing Respiratory Tract Infection in Children: A Systematic Review and Meta-Analysis. World Allergy Organ. J..

[88] De Boer G.M., Żółkiewicz J., Strzelec K.P., Ruszczyński M., Hendriks R.W., Braunstahl G.-J., Feleszko W., Tramper-Stranders G.A. (2020). Bacterial Lysate Therapy for the Prevention of Wheezing Episodes and Asthma Exacerbations: A Systematic Review and Meta-Analysis. Eur. Respir. Rev..

[89] Cao C., Wang J., Li Y., Li Y., Ma L., Abdelrahim M.E.A., Zhu Y. (2021). Efficacy and Safety of OM-85 in Paediatric Recurrent Respiratory Tract Infections Which Could Have a Possible Protective Effect on COVID-19 Pandemic: A Meta-analysis. Int. J. Clin. Pract..

[90] Yin J., Xu B., Zeng X., Shen K. (2018). Broncho-Vaxom in Pediatric Recurrent Respiratory Tract Infections: A Systematic Review and Meta-Analysis. Int. Immunopharmacol..

[91] Schaad U.B. (2010). OM-85 BV, an Immunostimulant in Pediatric Recurrent Respiratory Tract Infections: A Systematic Review. World J. Pediatr..

[92] Del-Rio-Navarro B.E., Espinosa-Rosales F.J., Flenady V., Sienra-Monge J.J. (2006). Immunostimulants for Preventing Respiratory Tract Infection in Children. Cochrane Database Syst. Rev..

[93] Zhang W., Huang J., Liu H., Wen X., Zheng Q., Li L. (2022). Whether Immunostimulants Are Effective in Susceptible Children Suffering From Recurrent Respiratory Tract Infections: A Modeling Analysis Based on Literature Aggregate Data. J. Clin. Pharma.

[94] Souza F.C.D., Mocellin M., Ongaratto R., Leitão L.A.D.A., Friedrich F.O., Silveira V.D., Scotta M.C., Pitrez P.M., Pinto L.A. (2020). OM-85 BV for Primary Prevention of Recurrent Airway Infections: A Pilot Randomized, Double-Blind, Placebo-Controlled Study. Einstein.

[95] Esposito S., Bianchini S., Bosis S., Tagliabue C., Coro I., Argentiero A., Principi N. (2019). A Randomized, Placebo-Controlled, Double-Blinded, Single-Centre, Phase IV Trial to Assess the Efficacy and Safety of OM-85 in Children Suffering from Recurrent Respiratory Tract Infections. J. Transl. Med..

[96] Sly P.D., Galbraith S., Islam Z., Holt B., Troy N., Holt P.G. (2019). Primary Prevention of Severe Lower Respiratory Illnesses in At-Risk Infants Using the Immunomodulator OM-85. J. Allergy Clin. Immunol..

[97] Chen J., Zhou Y., Nie J., Wang Y., Zhang L., Shi Q., Tan H., Kong W. (2017). Bacterial Lysate for the Prevention of Chronic Rhinosinusitis Recurrence in Children. J. Laryngol. Otol..

[98] Han R.-F., Li H.-Y., Wang J.-W., Cong X.-J. (2016). Study on Clinical Effect and Immunologic Mechanism of Infants Capillary Bronchitis Secondary Bronchial Asthma Treated with Bacterial Lysates Broncho-Vaxom. Eur. Rev. Med. Pharmacol. Sci..

[99] Lu Y., Li Y., Xu L., Xia M., Cao L. (2015). Bacterial Lysate Increases the Percentage of Natural Killer T Cells in Peripheral Blood and Alleviates Asthma in Children. Pharmacology.

[100] Liao J.-Y., Zhang T. (2014). Influence of OM-85 BV on hBD-1 and immunoglobulin in children with asthma and recurrent respiratory tract infection. Zhongguo Dang Dai Er Ke Za Zhi.

[101] Esposito S., Marchisio P., Prada E., Daleno C., Porretti L., Carsetti R., Bosco A., Ierardi V., Scala A., Principi N. (2014). Impact of a Mixed Bacterial Lysate (OM-85 BV) on the Immunogenicity, Safety and Tolerability of Inactivated Influenza Vaccine in Children with Recurrent Respiratory Tract Infection. Vaccine.

[102] Razi C.H., Harmancı K., Abacı A., Özdemir O., Hızlı Ş., Renda R., Keskin F. (2010). The Immunostimulant OM-85 BV Prevents Wheezing Attacks in Preschool Children. J. Allergy Clin. Immunol..

[103] Chen Z.-G., Ji J.-Z., Li M., Chen Y.-F., Chen F.-H., Chen H. (2007). Immunoregulants improves the prognosis of infants with wheezing. Nan Fang Yi Ke Da Xue Xue Bao.

[104] Gutiérrez-Tarango M.D., Berber A. (2001). Safety and Efficacy of Two Courses of OM-85 BV in the Prevention of Respiratory Tract Infections in Children During 12 Months. Chest.

[105] Jara-Pérez J.V., Berber A. (2000). Primary Prevention of Acute Respiratory Tract Infections in Children Using a Bacterial Immunostimulant: A Double-Masked, Placebo-Controlled Clinical Trial. Clin. Ther..

[106] Gómez Barreto D., De la Torre C., Alvarez A., Faure A., Berber A. (1998). Safety and efficacy of OM-85-BV plus amoxicillin/clavulanate in the treatment of subacute sinusitis and the prevention of recurrent infections in children. Allergol. Immunopathol..

[107] Collet J.P., Ducruet T., Kramer M.S., Haggerty J., Floret D., Chomel J.J., Durr F. (1993). Stimulation of Nonspecific Immunity to Reduce the Risk of Recurrent Infections in Children Attending Day-Care Centers. The Epicrèche Research Group. Pediatr. Infect. Dis. J..

[108] Paupe J. (1991). Immunotherapy with an Oral Bacterial Extract (OM-85 BV) for Upper Respiratory Infections. Respiration.

[109] Zagar S., Löfler-Badzek D. (1988). Broncho-Vaxom^®^ in Children with Rhinosinusitis: A Double-Blind Clinical Trial. ORL.

[110] Rosaschino F., Cattaneo L. (2004). Strategies for Optimizing Compliance of Paediatric Patients for Seasonal Antibacterial Vaccination with Sublingually Administered Polyvalent Mechanical Bacterial Lysates (PMBL). Acta Biomed..

[111] Bartkowiak-Emeryk M., Emeryk A., Roliński J., Wawryk-Gawda E., Markut-Miotła E. (2021). Impact of Polyvalent Mechanical Bacterial Lysate on Lymphocyte Number and Activity in Asthmatic Children: A Randomized Controlled Trial. Allergy Asthma Clin. Immunol..

[112] Emeryk A., Bartkowiak-Emeryk M., Raus Z., Braido F., Ferlazzo G., Melioli G. (2018). Mechanical Bacterial Lysate Administration Prevents Exacerbation in Allergic Asthmatic Children-The EOLIA Study. Pediatr. Allergy Immunol..

[113] Janeczek K., Kowalska W., Zarobkiewicz M., Suszczyk D., Mikołajczyk M., Markut-Miotła E., Morawska-Michalska I., Bakiera A., Tomczak A., Kaczyńska A. (2023). Effect of Immunostimulation with Bacterial Lysate on the Clinical Course of Allergic Rhinitis and the Level of γδT, iNKT and Cytotoxic T Cells in Children Sensitized to Grass Pollen Allergens: A Randomized Controlled Trial. Front. Immunol..

[114] Janeczek K., Emeryk A., Zimmer Ł., Poleszak E., Ordak M. (2022). Nasal Carriage of Staphylococcus Aureus in Children with Grass Pollen-Induced Allergic Rhinitis and the Effect of Polyvalent Mechanical Bacterial Lysate Immunostimulation on Carriage Status: A Randomized Controlled Trial. Immun. Inflamm. Dis..

[115] Bitar M.A., Saade R. (2013). The Role of OM-85 BV (Broncho-Vaxom) in Preventing Recurrent Acute Tonsillitis in Children. Int. J. Pediatr. Otorhinolaryngol..

[116] Cantarutti A., Barbieri E., Scamarcia A., Cantarutti L., Canova C., Giaquinto C. (2021). Use of the Bacterial Lysate OM-85 in the Paediatric Population in Italy: A Retrospective Cohort Study. Int. J. Environ. Res. Public Health.

[117] Lehtoranta L., Pitkäranta A., Korpela R. (2014). Probiotics in Respiratory Virus Infections. Eur. J. Clin. Microbiol. Infect. Dis..

[118] Vouloumanou E.K., Makris G.C., Karageorgopoulos D.E., Falagas M.E. (2009). Probiotics for the Prevention of Respiratory Tract Infections: A Systematic Review. Int. J. Antimicrob. Agents.

[119] OM Pharma SA (2025). A Randomised, Placebo-Controlled, 3-Arm, Double-Blind, Multicentre, Phase 4 Study to Assess the Efficacy of OM-85 (Broncho-Vaxom) Short- and Long-Term Treatment vs. Placebo in the Prevention of Respiratory Tract Infections in Children Aged Between 6 Months and 5 Years with Wheezing Lower Respiratory Illness. https://clinicaltrials.gov/study/NCT05677763.

[120] Tramper G. (2024). Protecting Late-Moderate Preterm Infants From Respiratory Tract Infections and Wheeze in Their First Years of Life by Using Bacterial Lysates. https://onderzoekmetmensen.nl/en/node/55914/pdf.

[121] Queen Mary University of London (2025). Oral Bacterial Lysate to Prevent Persistent Wheeze in Infants After Severe Bronchiolitis. A Randomised Placebo-Controlled Trial. https://www.menzies.edu.au/page/Research/Projects/Lungs/Oral_bacterial_lysate_to_prevent_persistent_wheeze_in_infants_after_severe_bronchiolitis_a_randomised_placebo-controlled_trial/.

[122] OM Pharma SA (2025). A Randomized, Placebo-Controlled, Double-Blind, Multicenter, Phase 2 Study to Assess the Efficacy and Safety of Daily OM-85 Treatment vs. Placebo Given in Children Aged 6 Months to 5 Years with Recurrent Wheezing. https://clinicaltrials.gov/study/NCT05857930.

[123] University of Arizona (2024). Randomized, Placebo-Controlled, Multicenter Study to Assess the Efficacy, Safety and Tolerability of ORal Bacterial EXtract for the Prevention of Wheezing Lower Respiratory Tract Illness (ORBEX). https://clinicaltrials.gov/study/NCT02148796.

[124] Bioithas S.L. (2025). Randomized, Double-Blind, Placebo-Controlled Clinical Trial to Evaluate the Efficacy and Safety of *Lacticaseibacillus rhamnosus*. CRL1505 in the Prevention of Upper Respiratory Tract Infections in a Healthy Paediatric Population.

[125] Castro-Rodriguez J.A., Turi K.N., Forno E. (2024). A Critical Analysis of the Effect of OM-85 for the Prevention of Recurrent Respiratory Tract Infections or Wheezing/Asthma from Systematic Reviews with Meta-analysis. Pediatr. Allergy Immunol..

[126] Mangino M., Roederer M., Beddall M.H., Nestle F.O., Spector T.D. (2017). Innate and Adaptive Immune Traits Are Differentially Affected by Genetic and Environmental Factors. Nat. Commun..

[127] Hernandez-Pacheco N., Kere M., Melén E. (2022). Gene-environment Interactions in Childhood Asthma Revisited; Expanding the Interaction Concept. Pediatr. Allergy Immunol..

[128] Hojsak I., Fabiano V., Pop T.L., Goulet O., Zuccotti G.V., Çokuğraş F.C., Pettoello-Mantovani M., Kolaček S. (2018). Guidance on the Use of Probiotics in Clinical Practice in Children with Selected Clinical Conditions and in Specific Vulnerable Groups. Acta Paediatr..

[129] Szajewska H., Vinderola G., Guandalini S., Indrio F. (2024). Current Regulatory Issues for the Use of Probiotics. Probiotics and Child Gastrointestinal Health: Advances in Microbiology, Infectious Diseases and Public Health Volume 19.

[130] Merenstein D.J., Tancredi D.J., Karl J.P., Krist A.H., Lenoir-Wijnkoop I., Reid G., Roos S., Szajewska H., Sanders M.E. (2024). Is There Evidence to Support Probiotic Use for Healthy People?. Adv. Nutr..

[131] Berber A., Del-Rio-Navarro B.E. (2019). Cost-Effectiveness Analysis of OM-85 vs Placebo in the Prevention of Acute Respiratory Tract Infections (ARTIs) in Children That Attend Day-Care Centers. Health Econ. Rev..

[132] Cunningham M., Azcarate-Peril M.A., Barnard A., Benoit V., Grimaldi R., Guyonnet D., Holscher H.D., Hunter K., Manurung S., Obis D. (2021). Shaping the Future of Probiotics and Prebiotics. Trends Microbiol..

[133] Huang X., Zhang X., Zhang Z., Liu M., Bai D., Yang R., Yang C. (2025). Bacterial Lysates in Allergic Rhinitis and Chronic Rhinosinusitis: Mechanisms and Clinical Evidence. Sci. Prog..

[134] Minute L., Montalbán-Hernández K., Bravo-Robles L., Conejero L., Iborra S., Del Fresno C. (2025). Trained Immunity-Based Mucosal Immunotherapies for the Prevention of Respiratory Infections. Trends Immunol..

