# Supplementary material for: Bacterial-Derived Immunomodulators as a Preventive Strategy for Viral Respiratory Tract Infections and Associated Wheezing or Asthma in Children: A Targeted Narrative Review
Source: Children (Basel). 2026 May 26;13(6):737. doi: 10.3390/children13060737 (PMC13297428; doi:10.3390/children13060737)
Supplement: Supplementary file 1 [file children-13-00737-s001.zip › children-4298780-supplementary.pdf]

ADDITIONAL FILE S1: SEARCH STRATEGIES AND METHODOLOGY

PART A: SEARCH STRATEGIES FOR PRECLINICAL EVIDENCE

1.OM-85

Search query:

- ("OM-85" OR "Broncho-Vaxom" OR "polyvalent bacterial lysate OM-85") AND ("mechanism of action" OR "immunomodulation" OR "immune response" OR "trained immunity" OR "immunometabolic") AND (preclinical OR "in vivo" OR "in vitro" OR experimental)
- ("OM-85" OR "Broncho-Vaxom") AND ("dendritic cells" OR "macrophages" OR "epithelial cells" OR "monocytes" OR "lymphocytes cells") AND ("activation" OR "signaling" OR "cytokines") AND ("in vitro" OR "cell line" OR "human cells")
- ("OM-85" OR "Broncho-Vaxom") AND ("mouse" OR "mice" OR murine) AND ("respiratory infection" OR "asthma model" OR "allergic airway" OR "influenza" OR "RSV") AND ("in vivo" OR "animal model" OR "murine model" OR preclinical)
- ("OM-85" OR "Broncho-Vaxom") AND ("sublingual" OR "oral") AND ("immune modulation" OR "inflammation") AND ("in vivo" OR "in vitro")
- ("OM-85" OR "Broncho-Vaxom") AND ("innate immunity" OR "adaptive immunity") AND ("immune modulation" OR "epithelial barrier") AND (preclinical OR "in vivo" OR 'in vitro")

Evidence Level: +++ (Extensive evidence)
Papers identified: 41 mechanistic studies
See additional file S2: Table S1 for complete list of studies

Studies span 42 years (1983-2025), demonstrating sustained research interest in OM-85 mechanisms. Studies include diverse experimental models: in vitro (epithelial cells, immune cells), in vivo (mouse models), and ex vivo (human samples).

2.PMBL (Ismigen)

Search query:

- ("PMBL" OR "Ismigen" OR "polyvalent mechanical bacterial lysate" OR "mechanical lysate") AND ("mechanism of action" OR "immunomodulation" OR "immune response" OR "mucosal immunity" OR "innate immunity") AND (preclinical OR "in vivo" OR "in vitro" OR experimental)
- ("PMBL" OR "Ismigen") AND ("dendritic cells" OR "macrophages" OR "epithelial cells" OR "monocytes") AND ("activation" OR "signaling" OR "cytokines") AND ("in vitro" OR "cell culture" OR "human cells")
- ("PMBL" OR "Ismigen") AND ("mouse" OR "mice" OR murine) AND ("respiratory infection" OR "RSV" OR "RV" OR "influenza") AND ("immune response" OR "immune activation") AND ("in vivo" OR "animal model" OR preclinical)
- ("PMBL" OR "Ismigen") AND ("lymphocytes" OR "T cells" OR "B cells" OR "Treg") AND ("in vivo" OR "in vitro")
- ("PMBL" OR "Ismigen") AND ("epithelial barrier" OR "cytokine" OR "immune cells") AND ("in vivo" OR "in vitro")

Evidence Level: + (Limited evidence)
Papers identified: 5 mechanistic studies
See additional file S2: Table S2 for complete list of studies

PMBL mechanistic evidence is primarily derived from clinical studies with immunological endpoints rather than traditional preclinical models. Studies span 2005-2023 and include both controlled trials and human volunteer studies with immune cell profiling.

3.MV130

Search query:

- ("MV130" OR "Bactek" OR "Bacmune") AND ("mechanism of action" OR "immunomodulation" OR "immune response" OR "trained immunity" OR "immunometabolic" OR "innate training") AND (preclinical OR experimental OR "in vivo" OR "in vitro")
- ("MV130" OR "Bactek" OR "Bacmune") AND ("dendritic cells" OR "monocytes" OR "macrophages" OR "epithelial cells") AND ("activation" OR "signaling" OR "cytokines") AND ("in vitro" OR culture OR "cell line" OR "human cells")
- ("MV130" OR OR "Bactek" OR "Bacmune") AND ("mouse" OR "mice" OR murine) AND ("trained immunity" OR "immune response" OR "respiratory infection" OR "asthma model" OR "allergic airway" OR "RV" OR "RSV" OR "influenza") AND ("in vivo" OR "animal model" OR "murine model" OR preclinical)
- ("MV130" OR "Bactek" OR "Bacmune") AND ("trained immunity" OR "epigenetic reprogramming" OR "metabolic shift") AND ("in vitro" OR "in vivo")
- ("MV130" OR "Bactek" OR "Bacmune") AND ("sublingual" OR "intranasal" OR "mucosal immunization") AND ("immune modulation" OR "lymphocytes" OR "T cells" OR "B cells" OR "Treg") AND ("in vivo" OR "in vitro")
- ("MV130") AND ("asthma" OR "allergic airway inflammation") AND ("IgE" OR "eosinophils") AND ("in vitro" OR "in vivo" OR "mouse model" OR "BAL)

Evidence Level: + (Limited evidence)
Papers identified: 5 mechanistic studies
See additional file S2: Table S3 for complete list of studies

MV130 research (2018-2025) focuses heavily on sublingual administration and trained immunity mechanisms. Studies combine murine models with human cell systems, emphasizing dendritic cell activation and metabolic reprogramming pathways.

4. Lactobacillus rhamnosus CRL1505

Search query:

- ("Lactobacillus rhamnosus CRL1505" OR "CRL1505") AND ("mechanism of action" OR "immunomodulation" OR "immune response" OR "innate immunity" OR "adaptive immunity") AND (preclinical OR "in vivo" OR "in vitro" OR experimental)
- ("Lactobacillus rhamnosus CRL1505" OR "CRL1505") AND ("macrophages" OR "dendritic cells" OR "epithelial cells" OR "intestinal epithelial cells") AND ("in vitro" OR "in vivo" OR "cell line")
- ("Lactobacillus rhamnosus CRL1505" OR "CRL1505") AND ("RSV" OR "influenza" OR "respiratory virus" OR "viral infection") AND ("immune response" OR "trained immunity") AND ("in vivo" OR "murine model" OR preclinical)

- (“Lactobacillus rhamnosus CRL1505” OR “CRL1505”) AND (“intestinal immunity” OR “gut mucosa” OR “epithelial barrier”) AND (“in vitro” OR “in vivo”)

Evidence Level: ++ (Moderate evidence)
Papers identified: 13 mechanistic studies
See additional file S2: Table S4 for complete list of studies

L. rhamnosus CRL1505 research (2020-2024) demonstrates strong focus on respiratory viral infections (RSV, rotavirus, influenza mimetics via poly(I:C)) and alveolar macrophage activation. Studies consistently employ combined in vitro/in vivo approaches, with particular emphasis on infant murine models and bacterial superinfection scenarios.

PART B: CLINICAL STUDIES

1.OM-85
Search query:

- OM-85 [Title/Abstract] AND (child* OR pediatric) AND (respiratory tract infection* OR RTI OR asthma) AND (randomized controlled trial [Publication Type] OR meta-analysis [Publication Type] OR systematic review [Publication Type])
- (Broncho-Vaxom OR Bronchovaxom) AND (child* OR pediatric) AND (respiratory infection* OR asthma OR RSV OR RV OR influenza) AND (randomized controlled trial [Publication Type] OR meta-analysis [Publication Type])

Results: 18 RCTs, 7 meta-analyses
See additional file S2: Table S5 for complete list of studies

2. PMBL
Search query:

- (PMBL OR Ismigen [Title/Abstract] AND (child*[Title/Abstract] OR pediatric [Title/Abstract]) AND (respiratory tract infection*[Title/Abstract] OR RTI[Title/Abstract] OR asthma [Title/Abstract]) AND (randomized controlled trial [Publication Type] OR meta-analysis [Publication Type] OR systematic review [Publication Type])
- "Polyvalent mechanical bacterial lysate"[Title/Abstract] AND (child*[Title/Abstract] OR pediatric [Title/Abstract]) AND (respiratory [Title/Abstract] OR asthma [Title/Abstract]) AND (randomized [Title/Abstract] OR meta-analysis [Publication Type])
- PMBL[Title/Abstract] AND (child* [Title/Abstract] OR pediatric [Title/Abstract]) AND (respiratory infection* [Title/Abstract] OR asthma [Title/Abstract]) AND (randomized controlled trial [Publication Type] OR clinical trial [Publication Type])
- ("recurrent respiratory infections"[Title/Abstract] OR "recurrent RTI"[Title/Abstract] OR "wheezing" [Title/Abstract]) AND (Ismigen [Title/Abstract] OR PMBL[Title/Abstract] OR "polyvalent mechanical bacterial lysate" [Title/Abstract]) AND (child* [MeSH Terms] OR pediatric [Title/Abstract])
- ("PMBL" [Title/Abstract] OR "mechanical lysate" [Title/Abstract]) AND Ismigen [Title/Abstract] AND child*[Title/Abstract] NOT OM-85[Title/Abstract]

Results: 5 RCTs, 1 meta-analysis
See additional file S2: Table S6 for complete list of studies

3. MV130
Search query:

- MV130 AND (child OR children OR pediatric) AND (respiratory tract infection OR RTI OR asthma) AND (randomized controlled trial [Publication Type] OR meta-analysis [Publication Type])
- MV130 AND (randomized controlled trial [Publication Type] OR meta-analysis[Publication Type])
- MV130 AND (child [Title/Abstract] OR children [Title/Abstract] OR pediatric [Title/Abstract]) AND ("RSV" OR "respiratory syncytial virus" OR "RV" OR "rhinovirus") AND (clinical trial [Publication Type] OR meta-analysis [Publication Type])
- ("MV130" OR "Bactek" OR "Bacmune" [Title/Abstract]) AND (child [Title/Abstract] OR children [Title/Abstract] OR pediatric [Title/Abstract]) AND (respiratory tract infection)
- ("MV130" OR "Bactek" OR "Bacmune" [Title/Abstract]) AND (child OR pediatric) AND (asthma OR wheezing OR "respiratory infection") AND ("meta-analysis" OR (randomized controlled trial)

Results: 1 RCT identified
See additional file S2: Table S7 for complete list of studies

4. *Lactobacillus rhamnosus* CRL1505
Search query:

- CRL1505 AND (child OR children OR pediatric) AND (respiratory tract infection OR RTI OR asthma) AND (randomized controlled trial [Publication Type] OR meta-analysis [Publication Type])
- CRL1505 AND (child [MeSH] OR child, preschool [MeSH] OR infant [MeSH] OR adolescent [MeSH]) AND (respiratory tract infections [MeSH] OR asthma [MeSH])
- "Lactobacillus rhamnosus CRL1505" AND (child OR children OR pediatric OR infant) AND ("respiratory syncytial virus" OR "rhinovirus" OR "influenza") AND (clinical trial OR randomized)
- "Lacticaseibacillus rhamnosus CRL1505" AND (randomized controlled trial [Publication Type] OR controlled clinical trial [Publication Type] OR meta-analysis [Publication Type])
- CRL1505 AND (clinical trial OR human OR children OR pediatric) AND ("respiratory infection" OR asthma)
- (CRL1505 OR "L. rhamnosus CRL1505" OR "Lactobacillus rhamnosus CRL1505") AND ("respiratory infection") AND (randomized controlled trial [Publication Type] OR meta-analysis [Publication Type])

Results: 0 pediatric RTI-specific RCTs identified

INCLUSION AND EXCLUSION CRITERIA

Inclusion Criteria:

- Pediatric population (0-18 years) for clinical studies
- Respiratory tract infections or wheezing/asthma as outcomes
- Original research articles
- Peer-reviewed publications
- English language

Exclusion Criteria:

- Duplicate publications
- Conference abstracts without full text
- Editorials and commentaries without original data
- Adult-only studies (for clinical evidence)
- Studies without respiratory outcomes

DATA EXTRACTION

For preclinical studies:

- Mechanism of action
- Cell types or animal models used
- Immunological pathways investigated
- Key findings

For clinical studies:

- Study design and population
- Intervention details (dose, duration, schedule)
- Primary and secondary outcomes
- Effect sizes and statistical significance
- Safety data

Search last updated: September 30, 2025
